# A soft body as a reservoir: case studies in a dynamic model of octopus-inspired soft robotic arm

**DOI:** 10.3389/fncom.2013.00091

**Published:** 2013-07-09

**Authors:** Kohei Nakajima, Helmut Hauser, Rongjie Kang, Emanuele Guglielmino, Darwin G. Caldwell, Rolf Pfeifer

**Affiliations:** ^1^Artificial Intelligence Laboratory, Department of Informatics, University of ZurichZurich, Switzerland; ^2^Bio-inspired Robotics Laboratory, Department of Mechanical and Process EngineeringETH Zurich, Zurich, Switzerland; ^3^Department of Advanced Robotics, Istituto Italiano di TecnologiaGenova, Italy; ^4^Key Laboratory of Mechanism Theory and Equipment Design of Ministry of Education, Tianjin UniversityTianjin 300072, P.R. China

**Keywords:** reservoir computing, octopus, soft robotics, morphological computation

## Abstract

The behaviors of the animals or embodied agents are characterized by the dynamic coupling between the brain, the body, and the environment. This implies that control, which is conventionally thought to be handled by the brain or a controller, can partially be outsourced to the physical body and the interaction with the environment. This idea has been demonstrated in a number of recently constructed robots, in particular from the field of “soft robotics”. Soft robots are made of a soft material introducing high-dimensionality, non-linearity, and elasticity, which often makes the robots difficult to control. Biological systems such as the octopus are mastering their complex bodies in highly sophisticated manners by capitalizing on their body dynamics. We will demonstrate that the structure of the octopus arm cannot only be exploited for generating behavior but also, in a sense, as a computational resource. By using a soft robotic arm inspired by the octopus we show in a number of experiments how control is partially incorporated into the physical arm's dynamics and how the arm's dynamics can be exploited to approximate non-linear dynamical systems and embed non-linear limit cycles. Future application scenarios as well as the implications of the results for the octopus biology are also discussed.

## 1. Introduction

Biological systems have certain morphologies^1^ and material characteristics that improve their adaptivity and increase their probability to survive. This suggests that control is not only located in the brain, but that there is a tight coupling between the brain, the body, and the environment, an idea that is usually termed *embodiment* (Pfeifer and Bongard, 2006; Pfeifer et al., 2007, 2012; Li et al., 2011b; Nakajima et al., 2011c, 2012a,b). Recently, motivated by the fact that soft material is ubiquitous in the body structures of living creatures, a new family of robots, *soft robots*, has been constructed with the aim of incorporating flexible elements (Trivedi et al., 2008; Steltz et al., 2009; Brown et al., 2010; Shepherd et al., 2011; Pfeifer et al., 2012). These robots have significant advantages over traditional articulated robots in terms of morphological flexibility and interactional safety (Trivedi et al., 2008; Li et al., 2011b). However, controlling them with conventional techniques is difficult because of their high-dimensional body structures and their diverse body dynamics, which are due to their non-linearity and elasticity. In this context, the octopus has been a good source of inspiration for roboticists to learn a control strategy for soft robots. An octopus has hyper-redundant limbs with a virtually unlimited number of degrees of freedom (DOF), and its movements are known to be highly sophisticated (Sumbre et al., 2001, 2005; Trivedi et al., 2008). From a conventional control perspective, the octopus's method of controlling movement and harnessing its non-linear body dynamics is outstanding and far-reaching.

It is well known that the nervous system of the octopus is highly distributed throughout the entire body. It has a relatively small central brain (about 50 million neurons), a central nervous system (CNS), which controls the large peripheral nervous system (PNS) of the arms (about 300 million neurons), integrates information from the visual system, and then issues commands to lower motor centers controlling the elaborate neuromuscular system of the arms. A typical example showing the effectiveness of this distribution of the nervous system is the reaching behavior (Gutfreund et al., 1996; Gutfreund, 1998; Sumbre et al., 2001; Yekutieli et al., 2005a,b). Reaching behavior consists of a *bend propagation* along the arm toward the tip in a highly stereotypical and invariant way. Sumbre et al. showed that the arm extensions can be evoked in arms whose connection with the brain have been severed (Sumbre et al., 2001). Because the evoked motions in denervated octopus arms were qualitatively and kinematically identical to natural bend propagations, an underlying motor program appears to be embedded in the neuromuscular system of the arm, which does not require continuous central control (Li et al., 2011a, 2012, 2013; Nakajima et al., 2011a,b; Kuwabara et al., 2012). In addition, the muscular organization of the octopus's arm has a characteristic structure called *muscular-hydrostats* (Kier and Smith, 1985; Smith and Kier, 1989; Taylor and Kier, 2003; Feinstein et al., 2011). In such structures, the volume of the organ remains constant during all movements, enabling the muscles themselves to perform all the functions usually performed by the skeleton (Sumbre et al., 2001, 2005; Taylor and Kier, 2003). This suggests that the body of the octopus arm is highly involved in the production of movements. Accordingly, in robotics, there have been several attempts to characterize the role of the muscular-hydrostat system in terms of its anatomical structure (Mazzolai et al., 2007; Laschi et al., 2009, 2012; Vavourakis et al., 2012a,b) and functionality (Nakajima et al., 2013).

In this paper, along the lines of these biological findings, we aim to provide one quantitative evidence that the structure of the octopus's arm has the potential to embed multiple motor programs without any support from the external controller. Recently, it has been shown that non-linear mass-spring networks can be used as a computational resource (Caluwaerts and Schrauwen, 2011; Hauser et al., 2011, 2012; Sumioka et al., 2011; Caluwaerts et al., 2013; Nakajima et al., 2013). These works imply that the non-linear and elastic body dynamics of soft robots are not drawbacks for control, but rather can be directly exploited as a computational resource. In this paper, we build on theoretical models (Hauser et al., 2011, 2012) that have been proposed in the context of *reservoir computing*.

The term reservoir computing has been proposed by Schrauwen et al. (2007) for a set of machine learning techniques used to emulate complex, non-linear computations. The idea is to drive a high-dimensional, non-linear dynamical system (which has been randomly initialized, but afterwards fixed) with a low-dimensional input stream. This dynamical system, typically referred to as the *reservoir*, provides highly complex, but reproducible responses in its state space to the input. It operates as a type of temporal and finite “kernel” by projecting non-linearily the low-dimensional input into the high-dimensional state space of the reservoir. Furthermore, since a reservoir consists of dynamical systems, it exhibits a memory, which fades out exponentially (i.e., fading memory)^2^. A remarkable property of the approach is, if the reservoir is complex enough (i.e., high-dimensional and non-linear), it can been shown that it is sufficient to add a simple *linear*, *static* readout from the high-dimensional state space to emulate *non-linear complex* computations. Such reservoir computing setups have been proven to outperform other machine learning techniques in a number of difficult tasks; see Jaeger (2003) for example. Another remarkable property of this setup is that the requested properties for computationally powerful reservoirs turn out to be rather general. Hence, a number of different implementations for reservoirs have been proposed (Schrauwen et al., 2007). For example, simple, abstract dynamical systems are used for echo state networks (Jaeger, 2002; Verstraeten et al., 2007; Lukoševičius and Jaeger, 2009), or models of neurons are used in liquid state machines (Maass et al., 2002). Lately, it has been demonstrated that complex, compliant bodies of biological systems and robots have the potential to serve as such a reservoir as well, see Hauser et al. (2011) and Hauser et al. (2012).

Here, we demonstrate that the soft robotic arm inspired by the octopus can be used as a reservoir. This means, by simply attaching a static, linear readout from the high-dimensional non-linear dynamics of the octopus arm, one can emulate complex, non-linear computation without altering the physical system itself. That is, we employ the physical body as part of a computational device. In this paper, a 3D dynamic model of this soft robotic arm is used as a test platform. Compared with the model used in Hauser et al. (2011) and Hauser et al. (2012), our model is more biologically inspired and physically feasible. It is a mass-spring-damper system, where the springs are aligned to emulate the octopus's muscular organization, and embeds the characteristic properties of a muscular-hydrostat. The arm is also assumed to be immersed in an underwater environment, in which the water friction constants are approximated by the computational fluid dynamics (CFD) simulations. As a result, the arm reveals highly non-linear body dynamics when actuated. By using this platform, we demonstrate that its body dynamics can be exploited as a computational resource. To test its power, we defined two types of tasks: first, to emulate complex non-linear dynamical systems, where we investigate whether the body dynamics are exploited as a computational resource; second, to implement a closed-loop control. We used several non-linear limit cycles to see how they can be embedded directly into the soft robotic arm without any support from an external controller. The choice of example functions adopted for each type of task is motivated to evaluate the non-linearity and memory that the body dynamics contains.

This paper is organized as follows. In section 2, we start by explaining the overall setting of the 3D dynamic model of the soft robotic arm platform and show how the arm emulates the muscular organization of the octopus in detail. The input–output relations adopted and the experimental procedures are also provided in detail. In section 3, we explain the results for a series of tasks in detail, and in section 4, we give concluding remarks, including future extension scenarios of our proposed approach.

## 2. Materials and methods

In this section, we provide a detailed description of the soft robotic arm simulator model and explain how to exploit the system as a computational resource by introducing input–output relations. The experimental procedure is also explained in detail.

### 2.1. Dynamic model of a soft robotic arm inspired by the octopus

**Figure 1:**
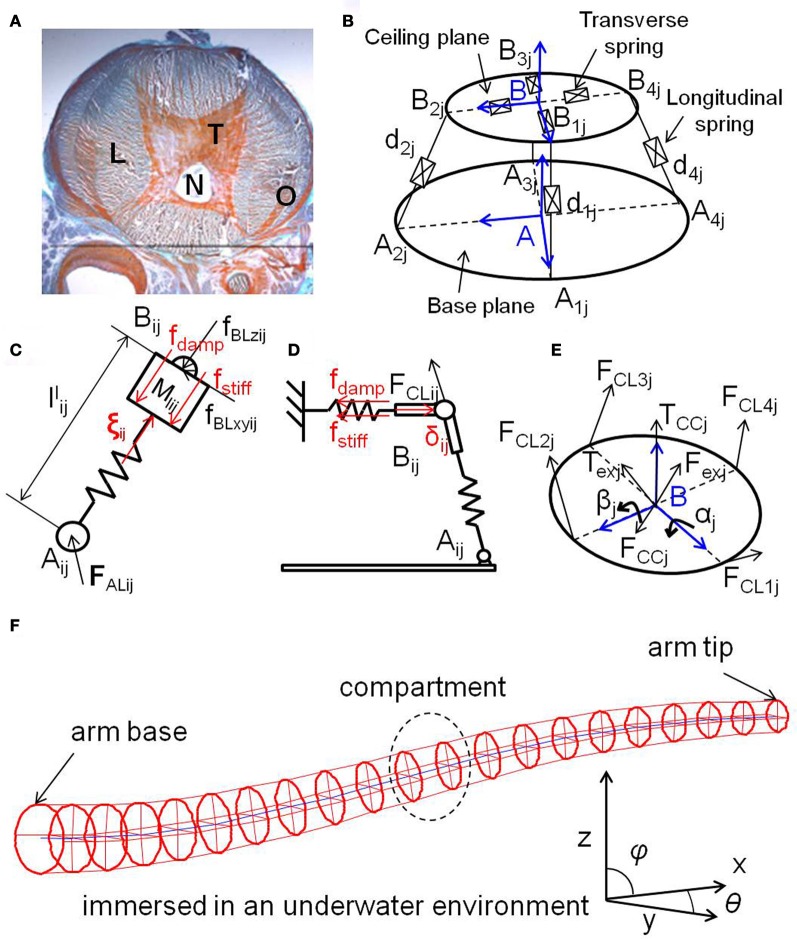
**(A)** Histological transverse section of an octopus arm, showing the longitudinal muscles (L), the transverse muscles (T), the nerve fibers (N), and the oblique muscles (O). **(B)** Overall structure of the muscular-hydrostat system (a single compartment) used in this paper. **(C)** Schematics showing a longitudinal spring, a transverse spring **(D)**, and a ceiling plane **(E)**. In **(C)**, $f_{\text{damp}}=C_{lij}{\dot{l}}_{ij}^{l},f_{\text{stiff}}=K_{lij}(l_{ij}^{l}-l_{0ij}^{l})$, and ξ_*ij*_ = ξ_*cij*_ + ξ_*vij*_. In **(D)**, $f_{\text{damp}}=C_{rij}{\dot{l}}_{ij}^{r},f_{\text{stiff}}=K_{rij}(l_{ij}^{r}-l_{0ij}^{r})$, and δ_*ij*_ = δ_*cij*_ + δ_*vij*_. See the text for details. **(F)** The entire soft robotic arm inspired by the octopus. It consists of 20 compartments, which are numbered from 1 to 20 from the base to the tip. Each compartment contains four longitudinal springs and one transverse disk. The blue line represents the line connecting the centers of each transverse disk. The base of the arm is able to rotate in any direction. See the text for details.

In this paper, we use a 3D dynamic model of a soft robotic arm inspired by the octopus (Kang et al., 2011, 2012). The model is currently applied for testing purposes for control architectures of soft robotic arms (Kuwabara et al., 2012; Nakajima et al., 2012a), and has been validated to have good agreement with a physical soft robotic arm platform (Zheng et al., 2012). The overall structure of the entire arm is shown in Figure 1F. It is assumed to be immersed in an underwater environment, and the base of the arm is able to rotate in any direction. It consists of 20 compartments, and each compartment implements the unique characteristics of octopus muscles, called muscular-hydrostats. In an octopus arm, muscles are organized into transverse, longitudinal, and obliquely oriented groups (Figure 1A). This special muscular organization forms the structures of the muscular-hydrostats. Their main property is that their volume remains constant during muscle contractions. The result is that if the diameter of the muscular-hydrostats decreases, then their length increases, and vice versa. Several proposed models deal with the muscular-hydrostat system of the octopus [e.g., See Yekutieli et al. (2005a,b) and Kang et al. (2012)]. The overall structure of the muscular-hydrostat system adopted in this paper is shown in Figure 1B. We begin our description by focusing on the model of a single compartment, and then progress to describing an entire arm.

A single compartment is a mass-spring-damper system, shaped like a circular truncated cone, consisting of a base plane, a ceiling plane with four transverse springs, a central strut, and four longitudinal springs, which emulate the anatomical structure of the muscle alignment in a real octopus arm. (Note that, although we use the term “spring,” it is a model for a muscle, so it has mass and damping.) The longitudinal springs control the position and orientation of the ceiling plane, while the transverse springs control the radius of the ceiling plane. The central strut provides kinematic constraints to guarantee the unique position of the ceiling plane. It is considered as an ideal prismatic joint without mass, damping, and stiffness. The system has an isovolmetric structure, which provides forces constantly aiming to maintain its volume and is an expression of the property of the muscular-hydrostats, and thus, all the springs are assumed to be implicitly or explicitly coupled with each other. The values for all the parameters of the model (e.g., spring coefficients, damping, etc.) are either inspired by the octopus or directly drawn from biological data (Kier and Curtin, 2002; Lieber, 2002; Shinohara et al., 2010). Standard SI units are used for the variables, and all of the ordinal differential equations presented are solved using the 4th order Runge–Kutta method, where *dt* is set to 0.001 *s* for the system throughout this paper.

Coordinates are defined on a base plane and a ceiling plane, denoted by *A*_*j*_(*x*_*j*_, *y*_*j*_, *z*_*j*_) and *B*_*j*_(*u*_*j*_, *v*_*j*_, *w*_*j*_), respectively (Figure 1B), where *j* is the index number of the compartment. A vector expressing a longitudinal spring (**d**_*ij*_) is: **d**_*ij*_ = **p**_*j*_ + **b**_*ij*_ −**a**_*ij*_, where $p_{j}=\bar{A_{j}B_{j}}={\left[0\;0\;h_{j}\right]}^{T}$ is the position vector of the center of the ceiling plane, **b**_*ij*_ is the position vector of joint *B*_*ij*_, and $a_{ij}=\bar{A_{j}A_{ij}}$ is the position vector of joint *A*_*ij*_. The length of the *i*th spring in compartment *j* is *l*^l^_*ij*_ and can then be obtained by $l_{ij}^{l}=\sqrt{d_{ij}^{T}\cdot d_{ij}}$. The dynamics of a longitudinal spring is expressed by:

(1) $$\xi_{cij}+\xi_{vij}-f_{\text{BL}zij}=M_{lij}{\ddot{l}}_{ij}^{l}+C_{lij}{\dot{l}}_{ij}^{l}+K_{lij}\left(l_{ij}^{l}-l_{0ij}^{l}\right),$$

where ξ_*cij*_ is the control force, ξ_*vij*_ is the isovolumetric constraint force, which will be also explained further in Equation (7), *f*_BL*zij*_ is the component force of joint *B*_*ij*_ acting along the spring *i* of compartment *j*, *l*^*l*^_0*ij*_ is the initial length of the spring, *M*_*lij*_ is the mass of the spring, *C*_*lij*_ is the damping coefficient, and *K*_*lij*_ is the stiffness coefficient (Figure 1C). Then, the rotation of the longitudinal springs can be formulated in frame *A* by:

(2) $$I_{ij}{\dot{\omega }}_{lij}=d_{ij}\times f_{{\text{BL}}_{xyij}},$$

where **f**_BL*xyij*_ is the component force of joint *B*_*ij*_ acting perpendicular to the spring, *I*_*ij*_ is the inertia moment of the spring about *A*_*ij*_, and ω_*lij*_ is the angular velocity of the spring about *A*_*ij*_, where ω_*lij*_ = (**d**_*ij*_/*l*^*l*^_*ij*_) × (**v**_*Bij*_/*l*^*l*^_*ij*_), and **v**_Bij_ is the velocity of *B*_*ij*_.

To interlink several compartments serially the reaction forces between the longitudinal springs and the base, **F**_*ALij*_, need to be calculated. These reaction forces are obtained by:

(3) $$F_{ALij}=M_{lij}{\overset{..}{d}}_{ij}-F_{\text{BL}ij},$$

where **F**_*ALij*_ is the joint force on *A*_*ij*_, and **F**_BL*ij*_ = **f**_BL*xyij*_ + **f**_BL*zij*_ is the joint force on *B*_*ij*_.

The four transverse springs spread around the central point of the ceiling plane. Figure 1D shows the illustration of a transverse spring. The dynamics of the length of the transverse spring are described as:

(4) $$\delta_{cij}+\delta_{vij}-F_{\text{CL}ij}\cdot u_{bij}=M_{rij}{\ddot{l}}_{ij}^{r}+C_{rij}{\dot{l}}_{ij}^{r}+\;K_{rij}\left(l_{ij}^{r}-l_{0ij}^{r}\right),$$

where δ_*cij*_ is the control force, δ_*vij*_ is the isovolumetric constraint force (which will be discussed in detail later), **F**_CL*ij*_ = −**F**_BL*ij*_ is the joint force of *B*_*ij*_ acting on the ceiling plane, **u**_*bij*_ is the unit vector of **b**_*ij*_, *M*_*rij*_ is the mass of the spring, *l*^*r*^_0*ij*_ is the initial radius of the ceiling plane, *C*_*rij*_ is the damping coefficient, and *K*_*rij*_ is the stiffness coefficient (Figure 1D). Figure 1E shows the illustration of the ceiling plane. The equation for the motion of the ceiling plane is:

(5) $$M_{\text{ce}ilj}{\left[0\;0\;{\ddot{h}}_{j}\right]}^{T}=F_{\text{CL}1j}+F_{\text{CL}2j}+F_{\text{CL}3j}+\;F_{\text{CL}4j}+F_{\text{CC}j}+F_{\text{ex}j},$$

where *h*_*j*_ is the height of the ceiling plane of compartment *j*, **F**_CC*j*_ = −**F**_BC*j*_ is the joint force on *B*_*j*_ acting on the ceiling plane, and **F**_ex*j*_ is the external force. The rotation is formulated as:

(6) $${}^{B_{j}}I_{\text{ceil}j}{\left[\overset{..}{\beta_{j}}\;\overset{..}{\alpha_{j}}\;0\right]}^{T}=\sum_{i\;=\;1}^{4}{}^{B_{j}}b_{ij}\times^{A_{j}}R_{B_{j}}^{T}F_{\text{CL}ij}+{\;}^{B_{j}}T_{\text{CC}j}{+}^{B_{j}}T_{\text{ex}j},$$

where ^*B*_*j*_^**T**_CC*j*_ is the constraint torque of joint *B*_*j*_ acting on the ceiling plane, ^*A*_*j*_^**R**^*T*^_*B*_*j*__ is the Euler rotation matrix, ^*B*_*j*_^**b**_*ij*_ is the position vector of *B*_*ij*_ expressed in frame *B*_*j*_, and ^*B*_*j*_^**I**_ceil*j*_ is the inertia matrix of the ceiling plane.

As explained previously, the system is isovolumetric due to its muscular hydrostat structure. This means that an increase in the longitudinal length will result in a reduction in the cross-sectional area and vice versa. A pair of antagonistic forces are applied to the longitudinal and transverse springs to guarantee the isovolumetric constraints. These are expressed as:

(7) $$\xi_{vij}=-K_{lv}\times |\delta_{cij}|\times (V_{cj}-V_{0j}),$$

(8) $$\delta_{vij}=-K_{rv}\times \left|\sum_{i\;=\;1}^{4}\xi_{ij}+F_{exj}\cdot p_{j}/h_{j}\right|\times (V_{cj}-V_{0j}),$$

where *V*_*cj*_ is the actual volume of compartment *j*, *V*_0*j*_ is the initial volume of the compartment, *K*_*lv*_ is the constraint force gain for the longitudinal springs, and *K*_*rv*_ is the constraint force gain for the transverse springs. From Equation (7), it can be seen that the constraint force ξ_*vij*_ is a function of the transverse spring force, δ_*cij*_, and the compartment volume change, *V*_*cj*_ − *V*_0*j*_. By applying ξ_*vij*_ to Equation (1), the longitudinal springs will act against the transverse springs to drive the volume change to zero. Similarly, another constraint force δ_*vij*_ is obtained by Equation (8) and applied to Equation (4) for the transverse springs to cancel the volume change induced by the longitudinal springs. Note that the external force, **F**_ex*j*_, is included in Equation (8) because it is acting on the compartment on joint *B*_*j*_, which may also change the length of the longitudinal springs and the volume of the compartment.

In addition to the forces generated by the muscles, typical external forces applied to the soft robotic arm in an underwater environment are gravity, buoyancy, and hydrodynamic forces. These are considered as distributed forces acting on each compartment as:

(9) $$\begin{array}{l} F_{exj}=F_{gj}+F_{bj}+F_{\text{hyd}j}\\ \;=F_{gj}+F_{bj}+\left(F_{\text{hydD}j}+F_{\text{hydL}j}\right), \end{array}$$

where **F**_ex*j*_ is the total external force acting on compartment *j*, **F**_*gj*_ is the gravity force, **F**_*bj*_ is the buoyancy force, and **F**_hyd*j*_ is the hydrodynamic force composed of the water drag force, **F**_hydD*j*_, and the water lift force, **F**_hydL*j*_.

The direction of buoyancy always opposes gravity. Thus, the resulting force due to gravity and buoyancy is:

(10) $$F_{gj}+F_{bj}=(\rho_{o}-\rho_{w})V_{cj}gu_{gj}=\rho_{o}V_{cj}g_{e}u_{gj},$$

where ρ_*w*_ is the density of water, ρ_*o*_ is the density of the octopus arm, *V*_*cj*_ is the volume of compartment *j*, **u**_*gj*_ is the unit vector indicating the direction of gravity for compartment *j*, *g* is the gravity constant, and *g*_*e*_ is the equivalent gravity constant defined as:

(11) $$g_{e}=\left(1-\frac{\rho_{w}}{\rho_{o}}\right)\text{​}g.$$

By adjusting the value of *g*_*e*_, both gravity and buoyancy forces are included in the model.

**Figure 2:**
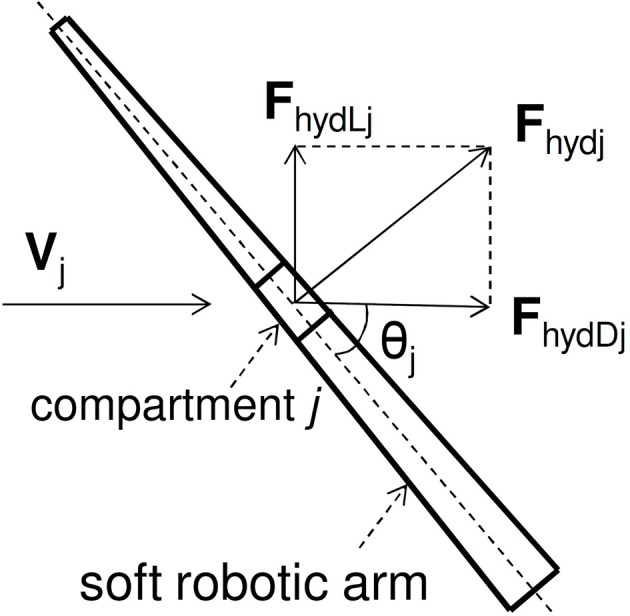
**Hydrodynamic forces acting on the soft robotic arm**.

The hydrodynamic forces applied to an octopus arm during a movement through a fluid medium are shown in Figure 2. For compartment *j*, the drag force **F**_hydD*j*_ is parallel to the velocity vector **V**_*j*_ of the fluid (or the uniform arm velocity in a stationary fluid) and the lift force **F**_hydL*j*_ is perpendicular to **V**_*j*_, according to

(12) $$F_{\text{hydD}j}=\frac{1}{2}C_{Dj}A_{rj}\rho_{w}||V_{j}|{|}^{2}u_{vj},$$

(13) $$F_{\text{hydL}j}=\frac{1}{2}C_{Lj}A_{rj}\rho_{w}||V_{j}|{|}^{2}u_{vj}^{⊥},$$

where *C*_*Dj*_ and *C*_*Lj*_ are the drag and lift coefficients, respectively, *A*_*rj*_ is the reference area of compartment *j*, **u**_*vj*_ is the unit vector indicating the direction of **V**_*j*_, and **u**^⊥^_*vj*_ is the unit vector of normal direction for **V**_*j*_. The hydrodynamic force coefficients, *C*_*Dj*_ and *C*_*Lj*_, for a segmented arm are obtained from high fidelity CFD simulations (Kazakidi et al., 2012). They were found to be dependent on the flow incidence angle θ_*j*_, and the configuration of the arm (e.g., straight vs. bended). As a first approximation of the hydrodynamic forces common in robotic literature (Ijspeert, 2001; Kazakidi et al., 2012), dependence on arm configuration was ignored. Therefore, a single value for each coefficient at specific angles of θ_*j*_ for a straight arm was identified by CFD simulations and approximated by a 4th order polynomial in the simulator, expressed as follows:

(14) $$C_{Dj}=e_{1}^{D}\theta_{j}^{4}+e_{2}^{D}\theta_{j}^{3}+e_{3}^{D}\theta_{j}^{2}+e_{4}^{D}\theta_{j}+e_{5}^{D},$$

(15) $$C_{Lj}=e_{1}^{L}\theta_{j}^{4}+e_{2}^{L}\theta_{j}^{3}+e_{3}^{L}\theta_{j}^{2}+e_{4}^{L}\theta_{j}+e_{5}^{L},$$

where *e*^*D*^_1−5_ and *e*^*L*^_1−5_ are the parameters identified by CFD simulations. The hydrodynamic forces for each compartment were then computed according to Equations (12) and (13), where **V**_*j*_ was taken as the velocity of *B*_*j*_. All the parameters used in this study are shown in Table 1.

**Table 1:** **Parameters for the soft robotic arm adopted in this paper**.

**Parameter**	**Value**	**Parameter**	**Value**
*l*^*l*^_0*ij*_ (m)	0.0200	*K*_*lij*_ (N/m)	196
*l*^*r*^_0*ij*_ (m)	0.015-0.0067	*K*_*rij*_ (N/m)	1570
*M*_*lij*_ (kg)	1.25 × 10^−3^	*K*_*lv*_ (l/m^3^)	1.0 × 10^6^
*M*_*rij*_ (kg)	1.25 × 10^−3^	*K*_*rv*_ (l/m^3^)	1.0 × 10^6^
*C*_*lij*_ (N s/m)	1.0	*V*_0j_ (m^3^)	1.38 × 10^−5^
			−2.99 × 10^−6^
*C*_*rij*_ (N s/m)	1.0	ρ_w_ (kg/m^3^)	1000
*e*^*D*^_1_	−5.5 × 10^−9^	*e*^*L*^_1_	1.8 × 10^−9^
*e*^*D*^_2_	6.3 × 10^−7^	*e*^*L*^_2_	−5.9 × 10^−7^
*e*^*D*^_3_	−7.7 × 10^−6^	*e*^*L*^_3_	2.8 × 10^−5^
*e*^*D*^_4_	0.0015	*e*^*L*^_4_	0.0011
*e*^*D*^_5_	0.017	*e*^*L*^_5_	0.00089

*Note that, for l^r^*_0*ij*_ and *V*_0*j*_, *they decrease monotonically, according to the compartment number in the given range in the table.*

This model is intrinsically non-linear. The non-linearities of the system are partly introduced by its kinematics (Kang et al., 2012). The relation between the spring length and the ceiling plane posture (position and orientation) is non-linear. Therefore, the system dynamics become non-linear as well. Also, the calculation of the isovolumetric forces and hydrodynamic forces introduces non-linearities. See Kang et al. (2012) for a detailed discussion of the model. In the majority of cases, these non-linearities are undesirable from the viewpoint of classical control theory. However, as previously mentioned in section 1, such a complex body could potentially be used as part of a computational device, if appropriate inputs and readouts are applied.

### 2.2. Experimental procedure

**Figure 3:**
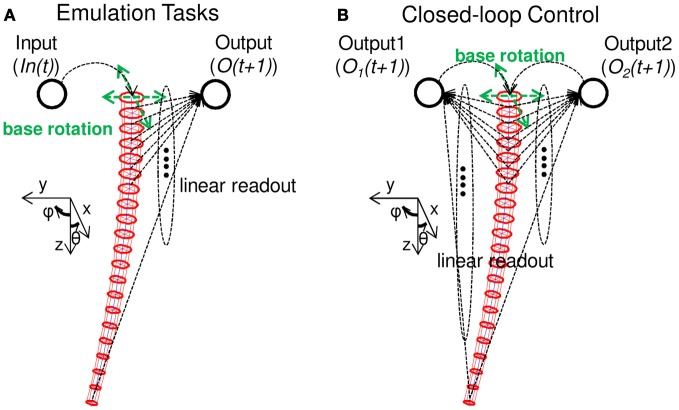
**Schematics explaining the tasks adopted in this paper. (A)** Schematics showing the nonlinear dynamical system emulation tasks (Task 1). An input (In(*t*)) is projected as a base rotation angle, and accordingly, the soft robotic arm shows passive body dynamics. By setting a linear readout for each longitudinal spring length in each compartment, an output (O(*t* + 1)) is calculated as a weighted sum of all the spring lengths. By adjusting only the linear readout, we demonstrate whether the system can emulate complex nonlinear dynamical systems. **(B)** Schematics showing the implementation of closed-loop control (Task 2). Similar to Task 1, by adjusting the linear readouts, two variables of a nonlinear limit cycle are emulated and fed back as the next input to the system to generate the base rotation movement. Accordingly, the nonlinear limit cycle is embedded onto the arm in a closed-loop manner. See the text for details.

Our aim in this paper is to demonstrate whether a soft robotic arm can be exploited as a computational resource, as well as a controller. Accordingly, we need to define inputs (In(*t*)) to the system and how to generate corresponding outputs (O(*t* + 1)). In this paper, we apply the position control of the base rotation as an input, and an output is generated by the weighted sum of the longitudinal spring lengths of all 20 compartments (Figure 3).

Based on this I/O scheme, we set two types of tasks for our demonstrations. First, we consider the emulation tasks of non-linear dynamical systems (Figure 3A), which aims to show whether the soft robotic arm can be exploited as a computational resource, including sufficient non-linearity and memory. The second task is to embed closed-loop control onto the soft robotic arm itself (Figure 3B). In particular, we aim to embed non-linear limit cycles, which are especially appealing for the control of robots. Typically, such limit cycles are implemented using non-linear oscillators, such as central pattern generators (CPGs), or a network of such oscillators (Righetti and Ijspeert, 2008). We here aim to demonstrate that the body of the soft robotic arm itself can be used to generate such limit cycles.

As explained in section 1, our approach is comparable to a reservoir computing approach, which normally uses randomly coupled non-linear elements as a computational resource (Jaeger, 2002; Maass et al., 2002; Jaeger and Haas, 2004). In the conventional reservoir computing approach, since each computational element is coupled randomly, each element possess a uniform role in the computation in the statistical sense. On the other hand, if we exploit the robot's body as a reservoir, according to the intrinsic structure of the body, each part of the body shows qualitatively different dynamics, which may lead to specific role distributions corresponding to each body part. Accordingly, in this paper, we will investigate how the computational role is distributed through the arm in each task. In the following subsections, we provide detailed descriptions for each task.

#### 2.2.1. Task 1: non-linear dynamical system emulation tasks

In order to evaluate the computational power of the system, we here set non-linear dynamical system emulation tasks, which are often used as benchmark tasks (Jaeger, 2002; Verstraeten et al., 2007; Hauser et al., 2011) in the context of recurrent neural network learning (Atiya and Parlos, 2000) and the reservoir computing approach (Jaeger, 2002; Maass et al., 2002; Jaeger and Haas, 2004). Each task requires a certain degree of non-linearity and memory to be performed by the system. As explained above, we first apply position control of the base rotation as an input (Figure 3A), expressed as follows:

(16) θ(t)=ϕ(t)=Scale×In(t),

(17) $$\text{In}(t)=0.2\sin(2\pi f_{1}t\times dt)\sin(2\pi f_{2}t\times dt)\sin(2\pi f_{3}t\times dt),$$

where θ(*t*) and ϕ(*t*) are base rotations at timestep *t* along the x-axis and y-axis, respectively. The parameter *Scale* linearly scales the raw input, In(*t*), to the specific range of the base angle [degree], −*R* ≤ θ(*t*), ϕ(*t*) ≤ *R*. This scaling parameter can be freely chosen, but should be fixed throughout the experiment. The detailed setting will be explained in the section 3. The parameters *f*_1_, *f*_2_, and *f*_3_ are set to 2.11, 3.73, and 4.33, respectively. Similar inputs were adopted in Hauser et al. (2011), Sumioka et al. (2011), and Nakajima et al. (2013).

According to the base rotation, the arm generates passive body dynamics. The output of the system is calculated by using the resulting spring dynamics as follows:

(18) $$\text{O}(t+1)=\sum_{j\;=\;1}^{20}\sum_{i\;=\;1}^{4}w_{\text{out}}^{ij}s_{ij}(t),$$

where *s*_*ij*_ (*t*) is the length of the longitudinal spring *i* (*i* = 1, 2, 3, and 4) in compartment *j* (*j* = 1, 2,…., 20) at timestep *t*. When the input is projected as the base rotation at timestep *t*, the corresponding length of the spring, *l*^*l*^_*ij*_, is collected to *s*_*ij*_ (*t*). The linear readout weight, *w*^*ij*^_out_, corresponds to each spring length. Overall, the dynamics of 80 (= 4 × 20) spring lengths are the expression of the body dynamics in this paper.

In order to achieve the required computation, we only train the linear readout (*w*^*ij*^_out_). Since we have 80 nodes, *s*_11_ (*t*), *s*_21_ (*t*), *s*_31_ (*t*), *s*_41_ (*t*), *s*_12_ (*t*),…., *s*_420_ (*t*), for the lengths of the spring at timestep *t*, by collecting the lengths of the springs for *M* timesteps, we can generate an 80 × *M* matrix **L**. We also collect the corresponding target outputs for *M* in a matrix **T**. Then, the optimal readout weights, **W** = [*w*^11^_out_, *w*^21^_out_, *w*^31^_out_, *w*^41^_out_, *w*^12^_out_,…., *w*^420^_out_]^*T*^, can be obtained by **W** = L^*^ **T**, where L^*^ is the Moore-Penrose pseudo-inverse, since **L** is not a square matrix in general.

According to the sent input, the system should emulate the following three non-linear dynamical systems as outputs. We prepared three corresponding output nodes to the system whose linear readouts are trained separately for each task. (This procedure is often called *multitasking*.) The first task is a 2nd order non-linear dynamical system, expressed as follows:

(19) $$y(t+1)=0.4y(t)+0.4y(t)y(t-1)+0.6{\text{In}}^{3}(t)+0.1,$$

where *y*(*t*) denotes the output of the system. The second task is a 10th order non-linear dynamical system, expressed as follows:

(20) $$y(t+1)=0.3y(t)+0.05y(t)\left(\sum_{i\;=\;0}^{9}y(t-i)\right)$$

(21)                 +1.5In(t−9)In(t)+0.1,

where *y*(*t*) denotes the output of the system. The third task is a discrete Volterra series, expressed as follows:

(22) $$y(t+1)=A\times \sum_{\tau_{1}=0}^{200}\sum_{\tau_{2}=0}^{200}h(\tau_{1},\tau_{2})\text{In}(t-\tau_{1})\text{In}(t-\tau_{2}),$$

(23) $$h(\tau_{1},\tau_{2})=\exp\text{​}\left(\frac{{\left(\tau_{1}\times dt-\mu_{1}\right)}^{2}}{2\sigma_{1}^{2}}+\frac{{\left(\tau_{2}\times dt-\mu_{2}\right)}^{2}}{2\sigma_{2}^{2}}\right)\text{​},$$

where *A* is a scaling parameter set to 0.0001, *y*(*t*) and *h* (τ_1_, τ_2_) denote the output of the system and a Gaussian kernel, respectively. The parameters, μ_1_, μ_2_, σ_1_, and σ_2_ are set as μ_1_ = μ_2_ = 0.1 and σ_1_ = σ_2_ = 0.05. Any computational model that can emulate the above dynamical systems should have a certain degree of memory and non-linearity. Simply put, emulation of a 10th order system requires more memory and non-linearity than a 2nd order system, and emulation of the Volterra task requires more than the 10th order system.

For the experimental procedure, the soft robotic arm is first set in the resting state with θ(*t*) = ϕ(*t*) = 0, and before beginning the experiment, we start to run the arm with Equation (16) for *T*_ini_ timesteps. This phase is to set the different initial positions of the arm for each experimental trial; *T*_ini_ is randomly determined from 0 to 1000 timesteps for each trial. The actual experimental trial consists of 16,000 timesteps, where the first 1000 timesteps are for washout, the following 10,000 timesteps are for the training phase, and the final 5000 timesteps are for the evaluation phase. After *T*_ini_ timesteps, we continue running the arm with Equation (16) and the actual experiment begins. By collecting the lengths of the spring and the corresponding target outputs for each task in the training phase, we train the linear readouts for three outputs by adopting the previously explained procedure. By using the trained linear readouts, we evaluate the performance of the system output by calculating the mean squared error (MSE), $\text{MSE}=\frac{1}{n}\sum_{t=1}^{n}{\left(\text{O(}t+1)-y(t+1)\right)}^{2}$, where *n* = 5000. We here compare the performance of the system with outputs generated by simple linear regression, O(*t* + 1) = *a* × In(*t*) + *b*, where *a* and *b* are trained by using the same time series as in the training phase. As is clear from the equation, since the linear regressor only uses the input to generate the output, which does not contain non-linearity and memory, any task performance of the system better than the linear regressor can be said that the required non-linearity and memory to perform the task is positively exploited from the system.

#### 2.2.2. Task 2: closed-loop control—embedding non-linear limit cycles

As previously explained, in this task, we aim to embed non-linear limit cycles in a closed-loop manner. The major difference from Task 1 is that the outputs generated by the system are fed back to the system itself as a motor command (an input) for the next timestep (Figure 3B). In particular, as will be explained later, we here aim to embed several limit cycles, which each have two variables. Accordingly, the outputs generated for the next motor commands (namely, In_1_(*t*) and In_2_(*t*) for each variable) are projected to θ(*t*) and ϕ(*t*), respectively. The situation is expressed as follows:

(24) $$\{\begin{array}{l} \theta (t)\text{​​​​​}={\text{In}}_{1}(t),\\ \phi (t)\text{​​​​​}={\text{In}}_{2}(t), \end{array}$$

(25) $$\{\begin{array}{l} {\text{In}}_{1}(t)\text{​​​​​}={\text{O}}_{1}(t),\\ {\text{In}}_{2}(t)\text{​​​​​}={\text{O}}_{2}(t), \end{array}$$

(26) $$\{\begin{array}{l} {\text{O}}_{1}(t+1)\text{​​​​​}=\sum_{j=1}^{20}\sum_{i=1}^{4}w_{\text{out},1}^{ij}s_{ij}(t),\\ {\text{O}}_{2}(t+1)\text{​​​​​}=\sum_{j=1}^{20}\sum_{i=1}^{4}w_{\text{out},2}^{ij}s_{ij}(t), \end{array}$$

where *w*^*ij*^_out, 1_ and *w*^*ij*^_out, 2_ are the linear readouts corresponding to the two outputs, O_1_(*t*) and O_2_(*t*), respectively. (Note that, in Equation (24), unlike Equation (16) in Task 1, the scaling parameter *Scale* is not introduced. As will be explained later, this is because we here aim to emulate the limit cycles, which are already scaled with a certain parameter value. So the scaling procedure is already included in the target outputs.)

As in the procedure explained in Task 1, to train the system, we only adjust the linear readouts, *w*^*ij*^_out, 1_ and *w*^*ij*^_out, 2_. However, the procedures differ in two points; first, during the training phase, we clamp the feedbacks from the system outputs, and provide the target outputs as inputs, which means, in Equation (25), we set In_1_(*t*) = *x*_1_(*t*), In_2_(*t*) = *x*_2_(*t*), where *x*_1_(*t*) and *x*_2_(*t*) are the target outputs at timestep *t*. Thus, the training phase is carried out with an open-loop, where the system was forced into the desired operative state by the target signals (this is called, *teacher forcing*) (Hauser et al., 2012). Second, when collecting the lengths of the springs in the training phase, we add white noise in the range of [−ν, ν]. By doing this, we can expect that the obtained optimal readouts will generate the target outputs even under the influence of noise (Hauser et al., 2012). The appropriate degree of ν is determined heuristically for each task.

Here, we aim to embed three non-linear limit cycles. The first one is the dynamical systems of the Van der Pol equations, expressed as follows:

(27) $$\{\begin{array}{l} \dot{x_{1}}\text{​​​​​}=x_{2},\\ \dot{x_{2}}\text{​​​​​}=-x_{1}+\left(1-x_{1}^{2}\right)x_{2}. \end{array}$$

The second one is a limit cycle, which we call the *quadratic limit cycle* (Hauser et al., 2012; Khalil, 2002), expressed as follows:

(28) $$\{\begin{array}{l} \dot{x_{1}}\text{​​​​​}=x_{1}+x_{2}-5x_{1}\left(x_{1}^{2}+x_{2}^{2}\right),\\ \dot{x_{2}}\text{​​​​​}=-2x_{1}+x_{2}-x_{2}\left(x_{1}^{2}+x_{2}^{2}\right). \end{array}$$

The third one is a Lissajous curve with a frequency ratio of *f*_1_/*f*_2_ = 2, expressed as follows:

(29) $$\{\begin{array}{l} x_{1}\text{​​​​​}=\sin(f_{1}t),\\ x_{2}\text{​​​​​}=\sin(f_{2}t). \end{array}$$

Since each limit cycle is symmetric about the point (0, 0), we select the variable with the larger range, and scale both variables to the desired range of the base rotation [degree], −*R* ≤ θ(*t*), ϕ(*t*) ≤ *R*. As in Task 1, this scaling parameter can be freely chosen, but should be fixed throughout the experiment. Thus, what the system should emulate here is *x*′_1_(*t* + 1) = Scale × *x*_1_(*t* + 1) and *x*′_2_(*t* + 1) = Scale × *x*_2_(*t* + 1) as O_1_(*t* + 1) and O_2_ (*t* + 1), respectively. Further settings on the parameter *Scale* will be explained in the section 3. For the Van der Pol system and the quadratic limit cycle, the ordinal differential equations are solved for each simulation timestep by using the 4th order Runge–Kutta method, where *dt* is set to 0.01. Note that the timescale of the arm model and these limit cycle is different. When we refer to time *s*, we always fixed our expression to the timescale to the arm model, otherwise we use the expression of simulation *timestep* to avoid the confusion.

In the experimental procedure, the soft robotic arm is first set in the resting state with θ(*t*) = ϕ(*t*) = 0, as in Task 1. We run the system with the teacher forcing condition for 70,000 timesteps, and by discarding the first 10,000 timesteps, we use 60,000 timesteps as for the training phase with white noise of degree ν added in the spring lengths. After obtaining the optimal readout weights from these collected data, we initialize the arm's state to the resting state and again start to run the system with the teacher forcing condition. After 5000 timesteps of running, we switch the inputs to the system output generated by the trained readout weights (Equations 25 and 26) and check whether it could embed the target limit cycle. Unlike Task 1, multitasking cannot be adopted (due to the feedback control), so each limit cycle is trained separately as a different trial.

## 3. Results

In this section, we present the results of each task applied to indicate the performance of our system. We would like to note again that all the tasks presented in this section is performed with “one body”, the same soft robotic arm explained in section 2.1, where all the parameter settings of the arm is fixed throughout the experiments. In addition, for Task 1, emulations of three non-linear dynamical systems are simultaneously performed for each experimental run (i.e., multitasking).

### 3.1. Task 1: non-linear dynamical system emulation tasks

**Figure 4:**
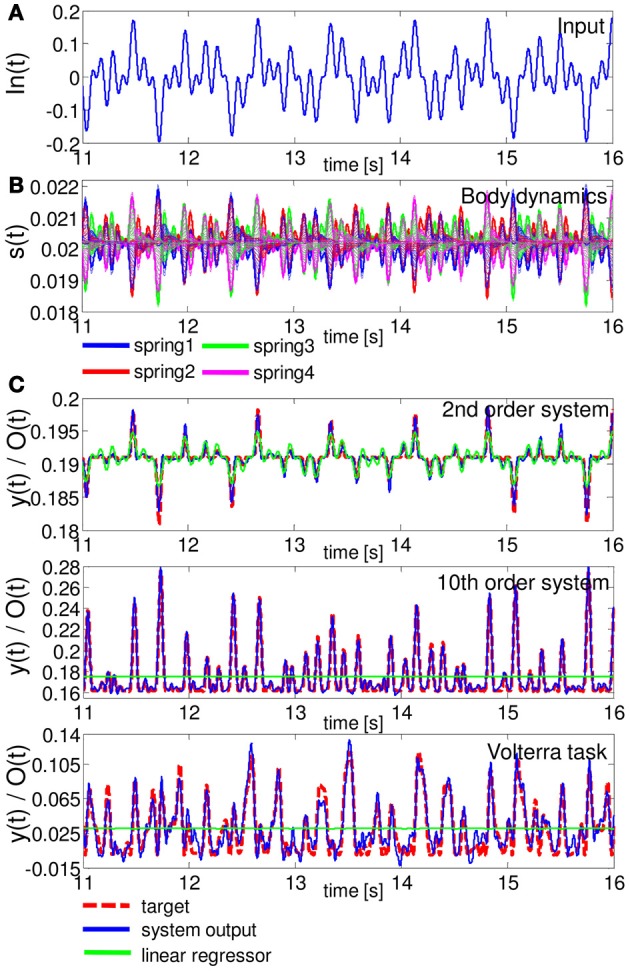
**A typical example of the task performance for Task 1 in terms of time series in the evaluation phase. (A)** The time series of In(*t*). **(B)** Corresponding body dynamics, which are expressed as the time series of the lengths of springs 1, 2, 3, and 4. The spring dynamics of all 20 compartments are overlaid. **(C)** Comparisons of the performance of the system output and the linear regressor for the emulation tasks of the 2nd order system (upper diagram), 10th order system (middle diagram), and Volterra task (lower diagram). In the plots, for the 2nd and 10th order systems, and the Voletrra task, the MSEs of the system output were 1.89 × 10^−7^, 3.80 × 10^−6^, and 8.90 × 10^−5^, respectively, and those of the linear regressor were 9.96 × 10^−7^, 4.95 × 10^−4^, and 9.48 × 10^−4^, respectively. For each task, the system output showed better performance than the linear regressor. Note that the output of the linear regressor is not a straight line, but a scaled version of the input with an offset (this outcome is due to the scaling of the figure).

Figure 4 shows a typical example of the time series of In(*t*), the lengths of the springs, and the performance of each task during the evaluation phase. The plots show the case when *R* is set to 60. We can clearly see that, according to the input projected to the base rotation, our soft robotic arm shows diverse passive body dynamics (Figures 4A,B). Regarding the task performance, the system output shows better performances than the linear regressor in all the tasks (Figure 4C). In the emulation task for the 2nd order system, the linear regressor also showed relatively good performance. However, as we can see in the plots, as the degree of non-linearity and memory of the task increases in the 10th order system and Volterra task, the performance of the linear regressor decreases significantly (Figure 4C). On the other hand, our system shows relatively good performance even in the Voletrra series emulation task (Figure 4C). Table 2 shows the statistical comparisons between the MSE of the output of the system and that of the linear regressor for each task. The values show the averaged MSEs over 20 trials. In each task, our system showed significantly low MSE. These results suggest that our system is able to exploit the non-linearity and memory originates from the passive body dynamics of the soft robotic arm to perform the task.

**Table 2:** **Comparisons of MSE between the output of the system and that of the linear regressor for each task**.

	**System output (*X*** ± ***SD*)**	**Linear regressor (*X*** ± ***SD*)**	***p* value**
2nd order	1.84 ± 0.05 (× 10^−7^)	1.01 ± 0.03 (× 10^−6^)	*p* < 0.001
10th order	3.77 ± 0.03 (× 10^−6^)	5.05 ± 0.11 (× 10^−4^)	*p* < 0.001
Volterra task	8.89 ± 0.18 (× 10^−5^)	9.28 ± 0.11 (× 10^−4^)	*p* < 0.001

**Figure 5:**
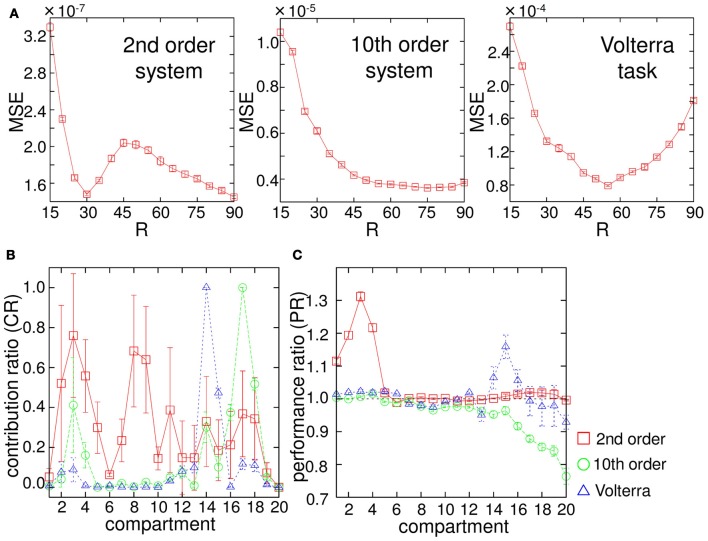
**(A)** Dependence of the performance of the system output (MSE) on the base rotation range, *R*, for each task. The plots show the averaged values of MSE over 20 trials. The error bars show the standard deviation. The averaged value of MSE of the linear regressor was much higher than that of the system output with respect to each *R* value (usually an order of magnitude higher). **(B)** Results of the contribution ratio analysis for each task. The horizontal axis shows the number of the compartment discarded in the evaluation phase, and the vertical axis shows the contribution ratio (CR). The plots show the averaged CR values over 20 trials, and the error bars show the standard deviations. **(C)** Results of the computational power analysis for each task. The horizontal axis shows the number of the compartment excluded to train the readout weights, and the vertical axis shows the performance ratio (PR). The plots show the averaged PR values over 20 trials, and the error bars show the standard deviations. See text for the details.

Unlike a conventional computational network or device, our system receives input as a mechanical rotation of the base, which generates the physical motion of the arm. Thus, we can easily imagine that, for example, if the input scaling of the base rotation range, *R*, is small, then the arm can only vibrate slightly, which does not generate diverse body dynamics. Moreover, since the input is linearly scaled to the base rotation range in our setting, the degree of this scaling changes not only the amplitude of the rotation but also the speed of the rotation. Considering that the water friction on the arm shows non-linear dependence on the velocity and the angle of the compartments (Equations 12, 13, and 14), the property of the body dynamics would change according to the degree of input scaling, *R*. Accordingly, the performance of our system would also change for each task. In order to validate this, we varied *R* from 15 to 90, and observed how the performance of the system changed for each *R*. Figure 5A shows the results. We can confirm the different individual error profile with respect to *R* for each task. First, small *R* values (around 15) show the highest errors; errors gradually start to decrease according to increases in *R* values in all tasks. But, for example, in the case of the 2nd order system, the error starts to increase again at around *R* = 30 and has a local maximum at around *R* = 45. In the case of the 10th order system, the error just decreases monotonically as the value of *R* increases. In the case of the Volterra task, the error shows the minimum at around *R* = 55 and start to increase monotonically as the value of *R* increases. This suggests that, even if the mechanical structure of the arm is the same, certain behaviors of the arm can reveal especially high computational power in some tasks, but not in others.

As explained in section 1, our system is essentially classified as a reservoir computing approach, in which a number of randomly coupled nodes are usually used as a computational resource, and where each node has a statistically uniform role. On the other hand, in our system, due to the intrinsic body structure, we can expect that there is a specific role for each body part. Here, we aim to investigate this point in two ways. First, when running the evaluation phase, we take out the readouts from one compartment and analyze the error. Note that, in this analysis, we use the readouts, which are trained with 20 compartments. By iterating this procedure for each compartment, we can investigate how each compartment contributed to the task performance when the readouts were fully connected. We call this *contribution ratio analysis* of the compartments. Second, we perform the entire experiment (i.e., washout, learning and evaluation phase) with only 19 compartments by skipping one compartment, and compare the performance with that obtained using 20 compartments. The difference with the previous contribution ratio analysis is that the readouts are, in the first place, trained to maximize the performance with 19 compartments including the entirely new readout weights. Since the readout weights are optimized without using a specific compartment, we can infer back the overall computational power of each compartment as a deviation from the original 20 compartment case. We call this *computational power analysis* of the compartments. Hereafter, the base rotation range, *R*, is fixed to 60 for the analyses.

In the contribution ratio analysis, the experimental procedure is the same as that explained in section 2.2, except that the evaluation phase is performed by taking out the readouts from a specific compartment (thus, four nodes are excluded). We iterate this procedure for each compartment by using the same input time series and body dynamics in a trial and calculate the MSE for each case. After testing all the compartments, we normalize them with the maximum MSE collected, and obtain *contribution ratio* (CR) for each compartment. If the CR is high for the compartment, then it implies that this compartment was contributing to the task performance largely when the readouts were fully connected. Figure 5B shows the result of the contribution ratio analysis for each task over 20 trials. In the case of the 2nd order system, although compartments 2, 3, 4, 8, and 9 seem to have high CRs, the standard deviations for these are also high, while compartments 1, 6, 19, and 20 have low CRs with low standard deviations. This suggests that specific compartments, such as 1, 6, 19, and 20 always contribute less to the task performance, while the computational role for this task is relatively distributed throughout the resting compartments, and among them, there is no specific compartment that consistently has a high contribution. In the case of the 10th order system and the Volterra task, the situation is different. There seems to be key compartments that always show high contributions to the task performance. In the case of the 10th order system, compartments 16, 17, and 18 show high performance, and in the case of the Volterra system, compartments 14 and 15 show high performance. Overall, these results suggest that our system adopts various strategies in the performance of computational abilities, according to the task. One strategy is to distribute the computational role throughout the entire arm, while the other is to always select and rely on the motion of specific body parts.

Next, in the computational power analysis, the experiment is performed both under the default condition (using 20 compartments) and with the exclusion of a specific compartment when training the readout weights. This is done by using the same input time series and body dynamics in a trial for each task. We calculated the MSEs in the evaluation phase for both cases and divided the MSE of the case without the specific compartment by that of the default condition and obtained the performance ratio (PR) for each task in each trial. Thus, if the PR is larger than 1.0, it implies that the task performance is worse than in the default condition, and the value indicates the degree of how the exclusion of the specific compartment affects the overall task performance in terms of ratio. Figure 5C shows the result of the computational power analysis for each task over 20 trials. In the 2nd order system task, we can clearly see that there are high PR values around the base of the arm (in compartments 1, 2, 3, and 4) suggesting that these compartments contain significant information for the performance of this task. Similarly, in the Volterra task, there are high PR values in compartments 14, 15, and 16. On the other hand, in the 10th order system task, PR values lower than 1.0 are shown around the tip of the arm (in compartment numbers higher than 15), suggesting that these compartments have a negative influence on the performance of this task. We speculate that this is caused by an overfitting effect produced by the compartments around the tip of the arm. The network is too specialized for the learning data and is not able to generalize to the new (evaluation) data. The reason for this should be explored in more detail in future work. Overall, we showed that there are specific regions in the body parts that contain positive or negative information for the performance of the tasks.

In this section, we first demonstrated that our soft robotic arm can perform the task to emulate non-linear dynamic systems by positively exploiting the non-linearity and memory originates from its body dynamics. We also confirmed that the way the input is applied (in our case, the amplitude range for the arm movement) significantly affects the computational ability, and its body parts show specialized roles due to their intrinsic morphological structure and corresponding diverse body dynamics, unlike the conventional reservoir computing approach. In the next section, we see how these body dynamics can potentially be used to control the arm's motion in a closed-loop manner by embedding non-linear limit cycles.

### 3.2. Task 2: closed-loop control—embedding non-linear limit cycles

In this section, we show the results for Task 2. By following the procedure described in section 2.2.2, we conducted a number of computer simulations to train the readouts with various values of the base rotation range *R* and the degree of white noise ν. As a result, we heuristically found that the system performance is extremely sensitive to the setting of these parameters [as opposed to the results presented for the simpler and abstract networks used in Hauser et al. (2012)]. (As for *R*, we have already shown in the previous section that *R* changes the computational power of the system significantly.) If these parameters were not set appropriately, we often observed that, when the system was switched from the teacher forcing condition to the closed-loop control, the arm gradually approached the resting state or showed unrealistic behaviors due to numerical problems. For the latter case, since we adopt the position control of the base angle, if the output showed much higher values than that of one timestep before (for example, if |O(*t* + 1) − O(*t*)| > 10, then the arm would have to rotate its base extremely quickly, namely, larger than 10^4^ deg/s, which is unrealistic in the physical platform), then, as a result, the simulator showed numerical problems. We carefully discarded these cases from our experiment. Even if the system has a high computational power as we saw in the previous section, the closed-loop setting requires additional care due to the stability issues. Since the output, which includes the error, is fed back to the system as input, the error may grow larger and larger in each simulation timestep.

**Figure 6:**
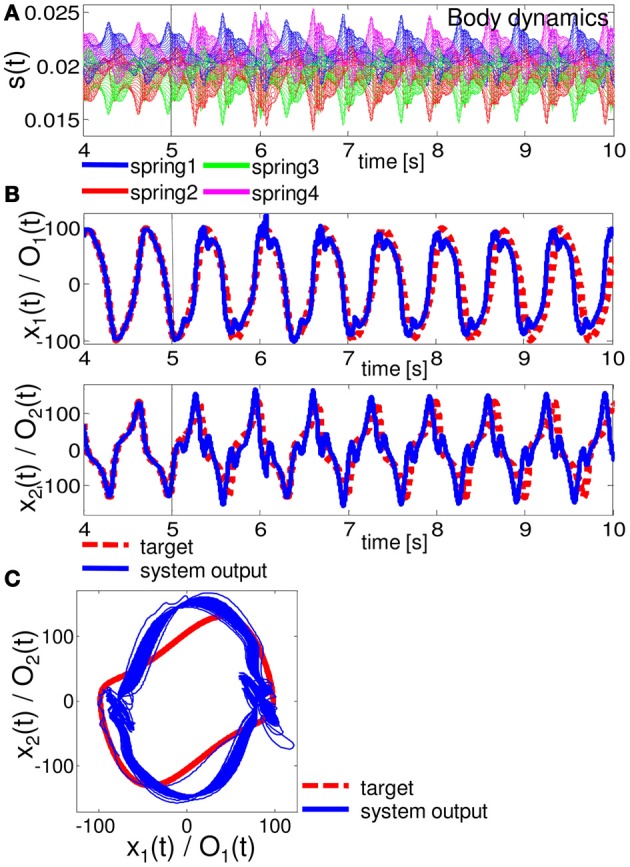
**Results for implementing the Van der Pol limit cycle.** At timestep 5000, the system is switched from the teacher forcing condition to the closed-loop control (black line). **(A)** The time series of the lengths of springs 1, 2, 3, and 4 for all 20 compartments. **(B)** Comparisons between the system output (O_1_ (*t*) and O_2_ (*t*) (blue lines)) and the target output (*x*_1_ (*t*) and *x*_2_ (*t*) (red lines)). **(C)** Comparisons between the system output (blue lines) and the target output (red lines) in the O_1_ (*t*) - O_2_ (*t*) (*x*_1_ (*t*) - *x*_2_ (*t*)) plane. The time series from timestep 5000 to timestep 70,000 are overlaid.

**Figure 7:**
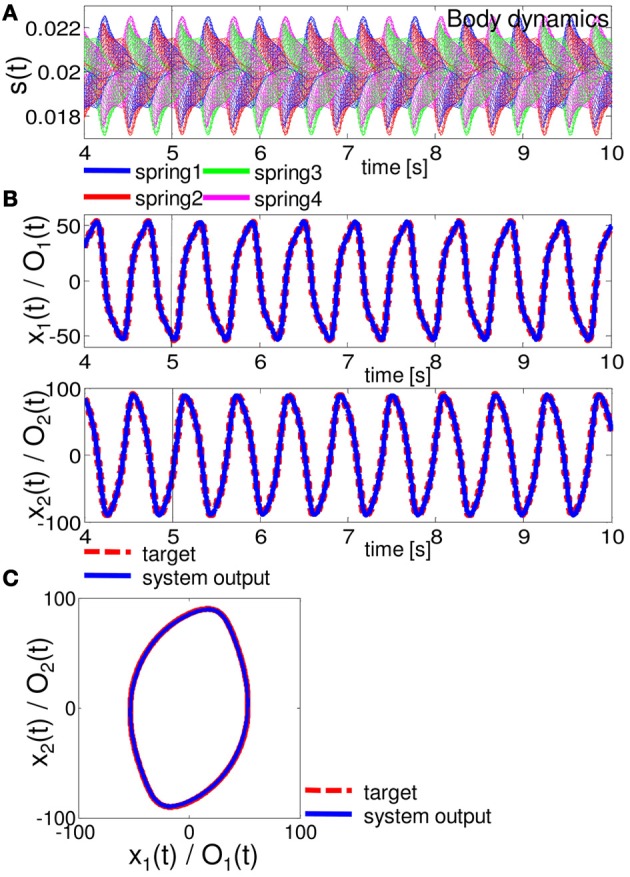
**Results for implementing the quadratic limit cycle.** At timestep 5000, the system is switched from the teacher forcing condition to the closed-loop control (black line). **(A)** The time series of the lengths of springs 1, 2, 3, and 4 for all 20 compartments. **(B)** Comparisons between the system output (O_1_ (*t*) and O_2_ (*t*) (blue lines)) and the target output (*x*_1_ (*t*) and *x*_2_ (*t*) (red lines)). **(C)** Comparisons between the system output (blue lines) and the target output (red lines) in the O_1_ (*t*) - O_2_ (*t*) (*x*_1_ (*t*) - *x*_2_ (*t*)) plane. The time series from timestep 5000 to timestep 70,000 are overlaid.

**Figure 8:**
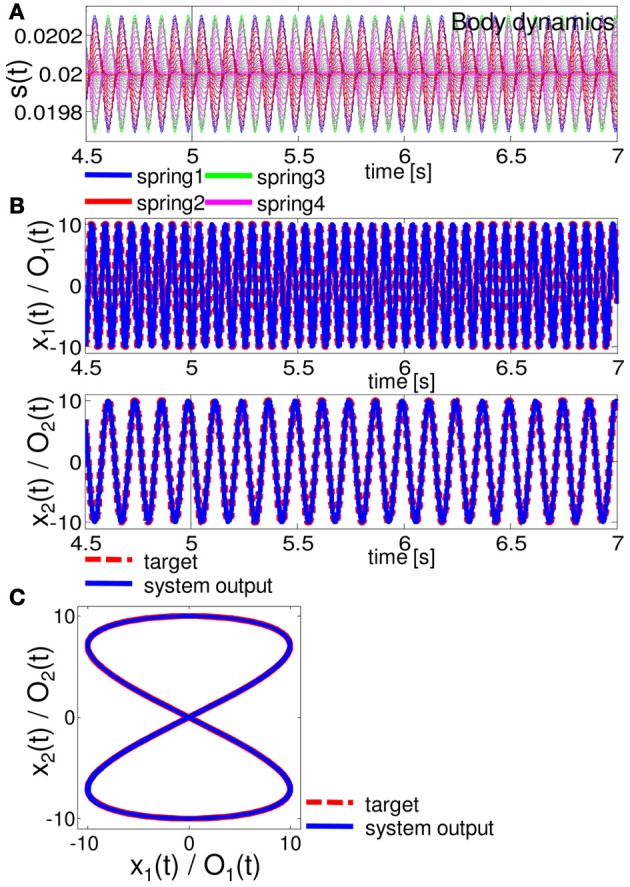
**Results for implementing the Lissajous curve.** At timestep 5000, the system is switched from the teacher forcing condition to the closed-loop control (black line). **(A)**. The time series of the lengths of springs 1, 2, 3, and 4 for all 20 compartments. **(B)** Comparisons between the system output (O_1_ (*t*) and O_2_ (*t*) (blue lines)) and the target output (*x*_1_ (*t*) and *x*_2_ (*t*) (red lines)). **(C)** Comparisons between the system output (blue lines) and the target output (red lines) in the O_1_ (*t*) - O_2_ (*t*) (*x*_1_ (*t*) - *x*_2_ (*t*)) plane. The time series from timestep 5000 to timestep 70,000 are overlaid.

Figures 6–8 show the typical results we obtained when the arm does not approach the resting state or the unrealistic behaviors mentioned above, for closed-loop control of the Van der Pol limit cycle, the quadratic limit cycle, and the Lissajous curve, respectively.^3^ For the parameters (*R*, ν), we adopted (*R*, ν) = (130, 1.5 × 10^−6^), (90, 1.0 × 10^−6^), and (10, 1.0 × 10^−11^), for the Van der Pol limit cycle, the quadratic limit cycle, and the Lissajous curve, respectively. For the Van der Pol limit cycle, we can see that the system is not implementing the target trajectory (Figures 6B,C), but is rather implementing an irregular one (Figure 6C). We also observed that the behavior of this trajectory is not as stable, but rather constantly changes its trajectory for each cycle, and this change remains throughout the trial. However, the results for the quadratic limit cycle and the Lissajous curve show almost a complete fit with the target trajectory (Figures 7B,C, and 8B,C, respectively). We confirmed that these trajectories were stable enough to run for 1,000,000 timesteps without leaving the trajectories of the target limit cycles. These results suggest that the task performance of the closed-loop control is not only restricted to the degree of non-linearity or memory required for the limit cycles but is also dependent on how the arm is driven. These preferences are caused by the intrinsic structure of the body. From now on, by using the system embedding the quadratic limit cycle (Figure 7) and the Lissajous curve (Figure 8), we move on to analyze the stability of the closed-loop controls and the role of each body part in these limit cycles as we saw in the previous section.

**Figure 9:**
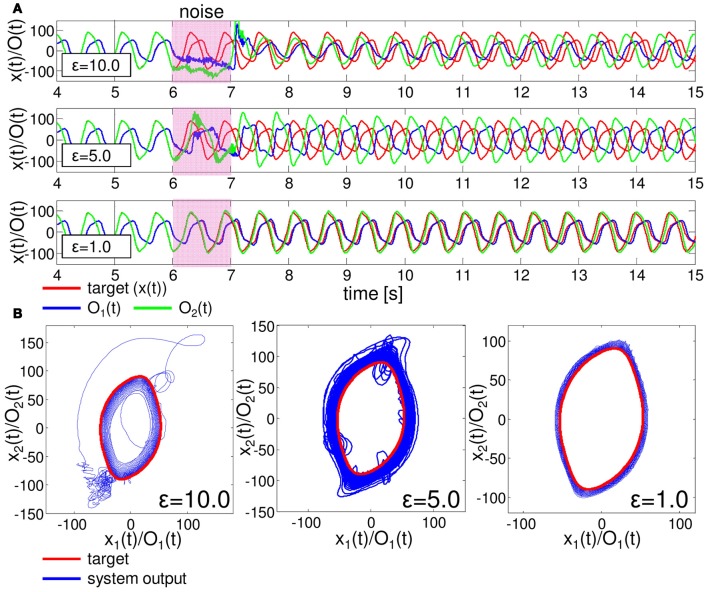
**Typical examples showing the performance of the embedded quadratic limit cycle with external noise.** The white noises, δO_1_ (*t*) and δO_2_ (*t*), in the range of ϵ (δO_1,2_ (*t*) ∈[−ϵ, ϵ]) are added to the motor outputs during *t* = 6000 − 7000 timesteps. **(A)** Typical trajectories of O_1_ (*t*) and O_2_ (*t*) compared with the target time series. The upper, middle, and lower figures show the case with ϵ = 10.0, 5.0, and 1.0, respectively. **(B)** The corresponding plots in the O_1_ (*t*) - O_2_ (*t*) (*x*_1_ (*t*) - *x*_2_ (*t*)) plane. For each noise level, the trajectories are overlaid from 5000 to 30,000 timesteps.

**Figure 10:**
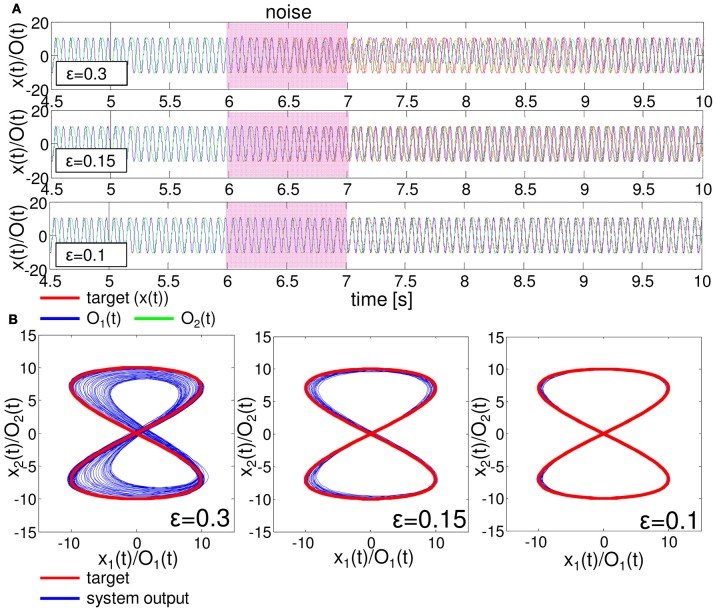
**Typical examples showing the performance of the embedded Lissajous curve with external noise.** The white noises, δO_1_ (*t*) and δO_2_ (*t*), in the range of ϵ (δO_1,2_ (*t*) ∈[−ϵ, ϵ]) are added to the motor outputs during *t* = 6000 − 7000 timesteps. **(A)** Typical trajectories of O_1_ (*t*) and O_2_ (*t*) compared with the target time series. The upper, middle, and lower figures show the case with ϵ = 0.3, 0.15, and 0.1, respectively. **(B)** The corresponding plots in the O_1_ (*t*) − O_2_ (*t*) (*x*_1_ (*t*) - *x*_2_ (*t*)) plane. For each noise level, the trajectories are overlaid from 5000 to 30,000 timesteps.

**Figure 11:**
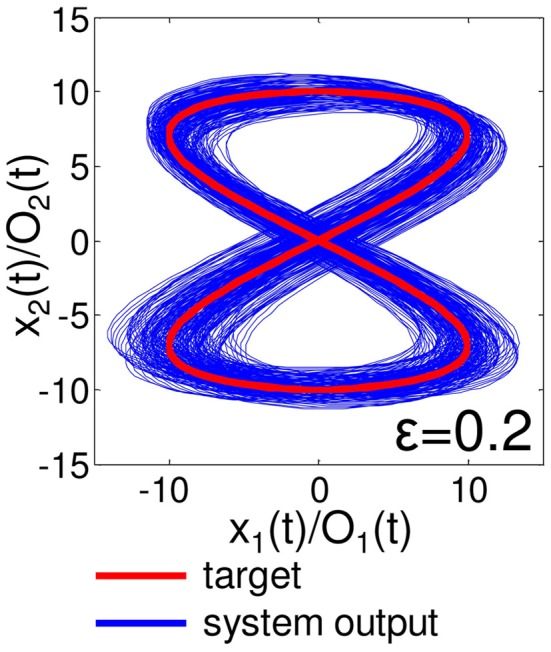
**Unstable trajectory of the embedded Lissajous curve with external noise in the range of ϵ = 0.2.** The plot is in the O_1_ (*t*) - O_2_ (*t*) (*x*_1_ (*t*) - *x*_2_ (*t*)) plane. The trajectories are overlaid from timestep 5000 to timestep 30,000. The target trajectory is also shown as a reference.

One important aspect to evaluate the embedded closed-loop control is its robustness against external perturbations. To test this, we added white noise, δO_1_ (*t*) and δO_2_ (*t*), in the range of ϵ (δO_1,2_ (*t*) ∈[−ϵ, ϵ]) to the two motor outputs of the embedded control, such as O_1_ (*t*) + δO_1_ (*t*) and O_2_ (*t*) + δO_2_ (*t*), during *t* = 6000–7000 timesteps for each as an example. Figure 9 shows the typical results of the performance of the embedded quadratic limit cycle regarding each noise level (ϵ = 10.0, 5.0, and 1.0). We can clearly see that even if the noise level is relatively large, such as ϵ = 10.0, the trajectories eventually recover to the limit cycle, which suggests that the embedded quadratic limit cycle is robust against external noise. We also confirmed that, even if we elongate the duration of time for the added noise, the system can successfully recover its performance. Note that, although the perturbed trajectories came back toward the limit cycle (Figure 9B), the oscillation phase was often shifted (Figure 9A). This was mainly caused by the relatively long duration of time for adding noise. We observed that by shortening this duration, this phase shift tendency can be reduced accordingly. Next, let us see the case for the embedded Lissajous curve. Compared to the quadratic limit cycle case, the system is less robust. When ϵ is more than around 0.3, we often observed that the trajectories go out from the limit cycle and never come back. Figure 10 shows the typical results of the performance of the embedded Lissajous curve for each noise level less than 0.3 (ϵ = 0.3, 0.15, and 0.1). If the noise level was less than around 0.3, we observed the system performance recovered toward the limit cycle as in the quadratic limit cycle case. However, even in this noise range, we sometimes observed an unstable trajectory as shown in Figure 11. In addition, similarly to the quadratic limit cycle case, even if the perturbed trajectories came back toward the limit cycle (Figure 10B), the oscillation phase was often shifted (Figure 10A).

Now, we move on to see the role of each body part (compartments or springs) as we saw in Task 1. Since the motor outputs and the body dynamics are reciprocally coupled through the feedback loop, the scheme we adopted in the Task 1 case, such as skipping one compartment, will cause unrealistic behavior of the arm due to numerical problems, as explained previously, and cannot always be adopted to appropriately evaluate the contributions of the body parts. Accordingly, we aim to investigate the contribution of each body part in terms of robustness against noise. Namely, we evaluate how the noise added to each body part affects the overall system performance. As is obvious from the system construction (Equations 24, 25, and 26) for the closed-loop control, the slight difference in the motor outputs at timestep *t* can affect the corresponding sensory time series, i.e., the lengths of the springs, and this effect influences the motor outputs at timestep *t* + 1. That is, according to the slight difference in the motor outputs, δO_1_ (*t*) and δO_2_ (*t*), the sensory time series, *s*_*ij*_ (*t*), will deviate from the original expressed as *s*′_*ij*_ (*t*) = *s*_*ij*_ (*t*) + δ*s*_*ij*_ (*t*), where *s*′_*ij*_ (*t*) is the actual spring length at timestep *t*. Then, from Equation (26), the outputs at timestep *t* + 1 can be simply expressed as:

(30) $$O^{\prime }(t+1)=\sum_{j=1}^{20}\sum_{i=1}^{4}w_{\text{out}}^{ij}s_{ij}^{\prime }(t),$$

(31) $$\;=\sum_{j=1}^{20}\sum_{i=1}^{4}w_{\text{out}}^{ij}\left(s_{ij}(t)+\delta s_{ij}(t)\right),$$

(32) $$\;=O(t+1)+\sum_{j=1}^{20}\sum_{i=1}^{4}w_{\text{out}}^{ij}\delta s_{ij}(t),$$

(33)              =O(t+1)+δO(t+1),

where O′(*t*) and O(*t*) are the actual and original motor outputs at timestep *t*, respectively. Note that for simplicity we dropped the index expressing two outputs. Since the deviation of the motor outputs is expressed as δO(*t* + 1) = ∑^20^_*j* = 1_ ∑^4^_*i* = 1_ *w*^*ij*^_out_δ*s*_*ij*_ (*t*), we can investigate how the noise applied to a single spring at timestep *t* can affect the motor outputs at timestep *t* + 1 by fixing the other springs as the original. By investigating how δO(*t* + 1) evolves through time, we can also evaluate the effect of the noise against the overall system performance.

**Figure 12:**
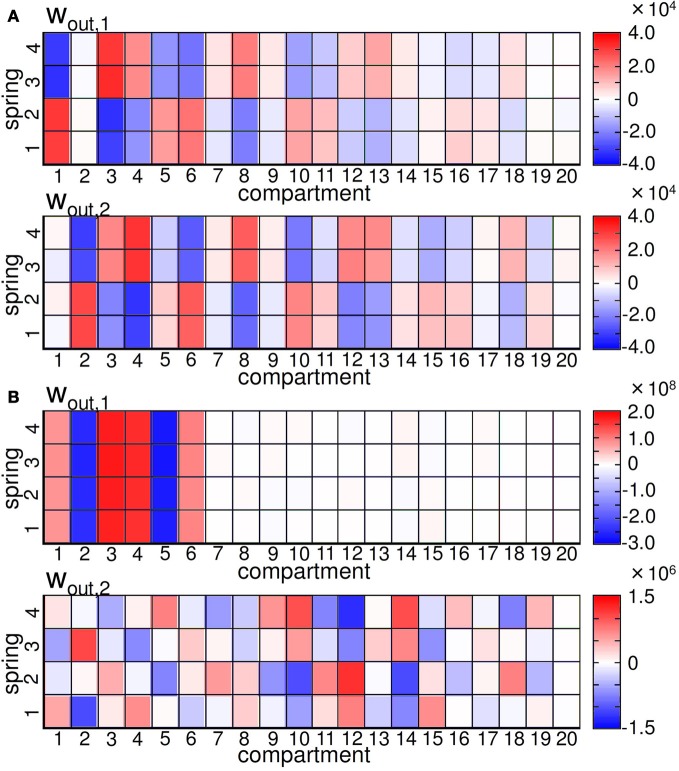
**(A)** Plots showing the readout weight distribution for the quadratic limit cycle. **(B)** Plots showing the readout weight distribution for the Lissajous curve. For each figure, the upper and lower graphs show *w*_out, 1_ and *w*_out,2_, respectively.

Now, let us assume that the noise was applied to the sensory value of the spring *i* in compartment *j* at timestep *t* by fixing the other sensory values as the original. Then, the deviation of the motor output at timestep *t* + 1 can be simply expressed as δO(*t* + 1) = *w*^*ij*^_out_δ*s*_*ij*_ (*t*), which straight-forwardly means that the degree of δO(*t* + 1) is only linearly dependent on the readout weight of the focused spring. Therefore, we can infer and compare how the noise added to each sensory value affects the motor output at timestep *t* + 1, regarding the fixed noise value, only by checking the weight distributions. Simply saying, if the value of |*w*^*ij*^_out_| is large, then the effect of the noise for spring *i* in compartment *j* at timestep *t* on the outputs for timestep *t* + 1 is also large, which means this spring makes a big contribution to the transition of the outputs for timestep *t* + 1. Figure 12 shows the readout weight distributions of *w*_out, 1_ and *w*_out, 2_ for the embedded quadratic limit cycle (Figure 12A) and Lissajous curve (Figure 12B). We can see that the distributions show a characteristic pattern for each limit cycle. For example, in the embedded quadratic limit cycle case, the value of the weights often seems to have a symmetric and corresponding distribution over springs in each compartment (e.g., between spring 1,2 and spring 3,4 in compartment 3–13), and even over *w*_out, 1_ and *w*_out, 2_ (Figure 12A). In the embedded Lissajous curve case, this type of symmetric and corresponding distribution can also be found within each weight, but not over *w*_out, 1_ and *w*_out, 2_ (Figure 12B). In addition, for the distribution of *w*_out, 1_, the value of the weights is almost zero in compartment 7–20, which means the external noise applied to these compartments at timestep *t* will not affect the motor outputs at timestep *t* + 1 very much.

**Figure 13:**
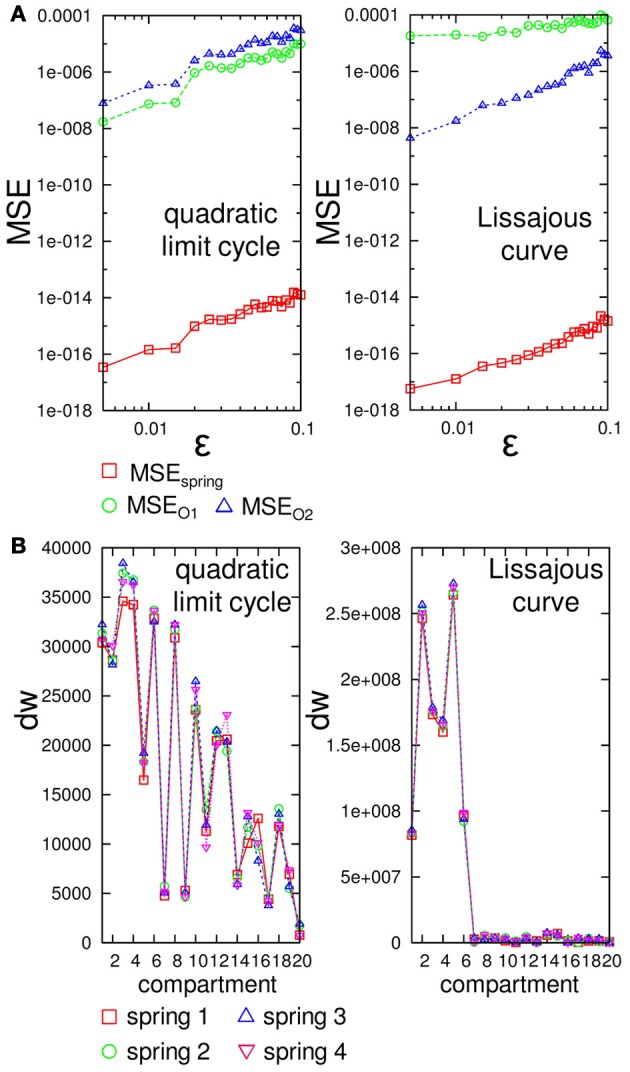
**(A)** Plots showing the averaged *MSE*_spring_, *MSE*_O1_, and *MSE*_O2_ according to each noise level ϵ for the embedded quadratic limit cycle (left) and Lissajous curve (right). Each MSE value is averaged over 15 trials by adding a randomly chosen value from [−ϵ, ϵ] to two outputs at timestep 6000. Note that both plots are in log-log plots. **(B)** Plots showing *dw* calculated by using the weights *w*_out, 1_ and *w*_out, 2_ for each spring *i* in each compartment for the embedded quadratic limit cycle (left) and Lissajous curve (right). See the text for details.

To systematically proceed with this line of analysis for the overall system performance, we need to confirm whether the large deviation in the sensory value at a specific time also leads to the large deviation of motor outputs over time. Although this seems to be trivial in our system, it is not trivial in general because the transition δO(*t*) → δ*s*_*ij*_ (*t*) depends on the construction of the body (for example, imagine the sensory value that exhibits a saturation)^4^. To evaluate this, we added a small white noise in the degree of ϵ to the motor outputs only at timestep 6000, and investigated how the differences in the motor outputs, |δO(*t*)|, and the spring lengths, |δ*s*_*ij*_ (*t*)|, carry on over time according to the degree of ϵ by measuring the mean square errors between the actual and original motor commands, such as ${\text{MSE}}_{\text{O}1}=\frac{1}{T}\sum_{t=1}^{T}{\left({\text{O}}_{1}^{\prime }(t)-{\text{O}}_{1}(t)\right)}^{2}$, ${\text{MSE}}_{\text{O}2}=\frac{1}{T}\sum_{t=1}^{T}{\left({\text{O}}_{2}^{\prime }(t)-{\text{O}}_{2}(t)\right)}^{2}$ and between the actual and original sensory time series as ${\text{MSE}}_{\text{spring}}=\frac{1}{80\times T}\sum_{t=1}^{T}\sum_{j=1}^{20}\sum_{i=1}^{4}{\left(s_{ij}^{\prime }(t)-s_{ij}(t)\right)}^{2}$, where *T* = 500 throughout this experiment. Note that we consider only a small range of noise around ϵ ∈ [0.005, 0.1], since if the noise level is too large, the trajectories often show phase shifts as we saw in the previous analysis (Figures 9, 10), which make the measures miss capturing the intended difference even if the trajectories were in the original limit cycles. Figure 13A shows the results of the averaged MSE_O_1, MSE_O_2, and MSE_spring_ for each embedded limit cycle. We can clearly confirm that according to the increase in the noise level ϵ, the value of each measure also increases, which means that the large deviation of the motor outputs at a specific time also leads to a large deviation in the motor outputs over time. In addition, in the embedded Lissajous curve case, MSE_O_1 is larger than MSE_O_2 for each ϵ value, which suggests that the output O_1_(*t*) is more sensitive than O_2_(*t*) (Figure 13A (right)).

Now, we are ready to investigate the role of each body part. According to the results shown in Figure 13A, the weights assigned for each spring directly reflect how the noise added to each sensory value affects the overall system performance. To correspond to the results in Figure 13A, we first calculated $dw=\sqrt{{\left(w_{\text{out},1}^{ij}\right)}^{2}+{\left(w_{\text{out},2}^{ij}\right)}^{2}}$ for each spring *i* in each compartment *j*, since the size of the noise to the motor output can be expressed as $\delta s_{ij}\sqrt{{\left(w_{\text{out},1}^{ij}\right)}^{2}+{\left(w_{\text{out},2}^{ij}\right)}^{2}}=\sqrt{\delta {\text{O}}_{1}{\left(t\right)}^{2}+\delta {\text{O}}_{2}{\left(t\right)}^{2}}\leq \epsilon$ by scaling the size of δ*s*_*ij*_ in the appropriate range. Thus, the value of *dw* directly reflects the contribution of each spring to the overall system performance regarding the fixed noise value. Figure 13B shows the value of *dw* for each spring *i* according to each compartment for the embedded quadratic limit cycle and Lissajous curve. Interestingly, the value of *dw* for each spring is almost the same within each compartment for both limit cycles, which means that the contribution of each body part can be expressed at the compartment level. In the case of the quadratic limit cycle, the value of *dw* shows almost a zig–zag pattern and gradually decreases when the compartment number increases from the base toward the tip [Figure 13B (left)]. In the case of the Lissajous curve, only the compartment around the base shows high values for *dw* [Figure 13B (right)]. These results suggest that, according to the limit cycles embedded, the sensitivity and degree to affect the overall system performance against the noise show different tendencies for each body part.

In this section, we have investigated whether our soft robotic arm can embed limit cycles in a closed-loop manner, and have shown that several properties, such as the system performance, the robustness against external noise, and the role of each body part differ according to which limit cycle to embed. Since we are adjusting only the linear readouts by using the same body, we can speculate that these specific properties corresponding to each limit cycle are caused by the intrinsic body structure.

## 4. Discussion

In this paper, by using the dynamic simulator of the soft robotic arm inspired by the octopus, we demonstrated that the robot's body dynamics are already capable of emulating non-linear dynamical systems and embedding non-linear limit cycles in a closed-loop manner by only adjusting the fixed linear readouts. The arm we used did not contain any rigid components. Instead, it is soft, including only springs, which are aligned to mimic the muscular structure of the octopus. This resulted in several compartments, each of which had a specific muscular-hydrostat property, which enforced the springs to be coupled in well-defined, but constrained, manner. In addition, the arm was assumed to be immersed in an underwater environment, in which the friction constants were identified via CFD simulations. All these factors, including this intrinsic body structure and its interaction with the environment, generated diverse body dynamics, including rich non-linearity and memory. The technique presented here allowed us to exploit these properties as computational resources. In addition, it is possible to infer the amount of non-linearity and memory that can be potentially exploited for information processing in terms of the task performance. For roboticists, this may open up the way to quantitatively characterize which control is efficient for which body design, as well as outsourcing the control load to the body parts. Although we kept the arm's mechanical structure as bio-inspired as possible throughout the analyses, it would also be meaningful to investigate how the information processing capability would change if the arm's mechanical properties (such as stiffness, damping, drag parameters) are altered. This line of experimentation will be included in our future work.

For the emulation tasks of non-linear dynamical systems, in addition to its high computational power, we showed that each body part has a specific role according to the task type. Additionally, for the closed-loop control tasks, we showed that the arm prefers some limit cycles over others (i.e., the quadratic limit cycle and the Lissajous curve were possible to embed, while the Van der Pol limit cycle was not). These obvious and specific coherencies are usually not observed in conventional reservoir computing where the reservoir consists of randomly coupled non-linear computational elements, suggesting that these properties originate from the intrinsic body structure.

From a biological systems point of view, this result seems natural. In nature, animals adapt to their respective ecological niches, where they evolve their body morphology to survive within their environment. The octopus is not an exception; its specific body structure is specialized to permit survival in a complicated underwater environment, enabling it to behave efficiently in particular ways. In this context, it would be interesting to investigate whether the arm could embed more biologically plausible behaviors in future work. For example, as we mentioned earlier, it is well known that the octopus adopts a specific strategy for reaching, called *bend propagation* (Gutfreund et al., 1996; Gutfreund, 1998; Sumbre et al., 2001; Yekutieli et al., 2005a,b). In this specific motion, it is suggested that the CNS only initiates the motion and all the muscle activations are handled at the PNS level (Gutfreund et al., 1996; Gutfreund, 1998; Sumbre et al., 2001). Several researches have investigated this behavior by directly extracting the muscle contraction patterns from the real octopus, and by externally applying these patterns to the octopus arm models (Gutfreund et al., 1996; Gutfreund, 1998; Yekutieli et al., 2005a,b). On this point, our technique presented here may reveal further insights on this overall scheme by including the role of the arm's body dynamics. Considering that the PNS does not have a plasticity (Kandel et al., 2000), it would be worth investigating how the arm's body dynamics, together with the PNS, modeled as a linear and static feedback loop onto the arm, embeds the motor patterns of bend propagation according to the initiation command sent by the CNS. This line of experiment can be investigated in future work.

There exists a growing number of documented cases in nature, which support that certain morphologies found in animals are facilitating a kind of computation. This observation is usually characterized by the term *morphological computation*. For example, the non-linear, non-homogeneous spatial arrangement of the ommatidia in insect eyes are more dense toward the front than on the side in order to compensate for motion parallax, which is non-linear (Franceschini et al., 1992). The morphology counteracts the non-linearity introduced by the parallax; hence, the complexity of the computational tasks to steer through obstacles based on the visual input is reduced. Since the resulting task for the brain is now simpler and not non-linear anymore due to the “clever” morphology, one could argue that part of the computation is conducted by the morphology. While this is a very simple case of a morphological computation, since the given morphology represents only a static, non-linear mapping, the concept does go further, if we consider, for example, soft, compliant bodies. Such bodies exhibit interesting dynamic properties, such as fading memory and non-linearity. Examples of such complex computations outsourced to the physical layer are passive walkers (Collins et al., 2005). Their design pushes the limits of what can be outsourced to the physical body, in so far that no controller (i.e., CPU) is needed at all. The mechanical design inspired by the musculoskeletal structure enabling “preflexes”, which can self-stabilize movements through its elastic material properties, also gives such an example (Brown et al., 1995; Blickhan et al., 2007; Proctor and Holmes, 2010). Their morphology (i.e., the mechanical, soft design and the environment) is able to “do” all the computations needed to walk robustly. While such robots are impressive, their disadvantage is their inflexibility by restricting the computation to a fixed physical body only. In a biological system, a sensible distribution of the computation between the body and the brain is more probable. Despite the number of biological examples and the series of robots, which have been built considering the concept of morphological computation in their design, there are still a few studies characterizing the concept within a quantitative framework (Hauser et al., 2011, 2012; Füchslin et al., 2013). In this context, we believe that the approach presented here would be one of the interesting directions for further study.

## Funding

This work was supported by the European Commission in the ICT-FET OCTOPUS Integrating Project (EU project FP7-231608), and was partially supported by JSPS Postdoctoral Fellowships for Research Abroad.

### Conflict of interest statement

The authors declare that the research was conducted in the absence of any commercial or financial relationships that could be construed as a potential conflict of interest.

## Footnotes

^1^By morphology, we do not only refer to the shape, but also sensor and actuator distributions, and physical properties, such as stiffness, etc.

^2^The memory is due to the integration capability of dynamical systems (i.e., It accumulates information over time)

^3^Strictly speaking, to prove that the embedded trajectory is really a “limit cycle”, we need to analytically show whether the trajectory is an attractor of the system. In our case, this is unrealistic because the equation governing the mechanical system is too complex, and we would have to rely on a heuristic approach. Accordingly, as we see later, we here call the embedded trajectory a “limit cycle” if and only if the trajectory can stay at the target limit cycle for 1,000,000 timesteps and the trajectory has a certain attraction when perturbed externally.

^4^Theoretically, this is to analyze the basin structure surrounding the original trajectory. Due to the number of parameters, instead of analyzing the basin structure according to δO_1_(*t*) and δO_2_(*t*), we analyzed the basin volume in terms of the error measures introduced later according to |δO(*t*)|.

## References

[B1] Atiya A. F. Parlos A. G. (2000). New results on recurrent network training: unifying the algorithms and accelerating convergence. IEEE Trans. Neural Netw. 11, 697–709 10.1109/72.846741 18249797

[B2] Blickhan R. Seyfarth A. Geyer H. Grimmer S. Wagner H. Gunther M. (2007). Intelligence by mechanics. Philos. Trans. R. Soc. A Math. Phys. Eng. Sci. 365, 199–220 10.1098/rsta.2006.1911 17148057

[B3] Brown E. Rodenberg N. Amend J. Mozeika A. Steltz E. Zakin M. R. (2010). Universal robotic gripper based on the jamming of granular material. Proc. Natl. Acad. Sci. U.S.A. 107, 18809–18814 10.1073/pnas.1003250107

[B4] Brown I. E. Scott S. H. Loeb G. E. (1995). Preflexes – programmable, high-gain, zero-delay intrinsic responses of perturbed musculoskeletal systems. Soc. Neurosci. Abst. 21, 562.9

[B5] Caluwaerts K. D'Haene M. Verstraeten D. Schrauwen B. (2013). Locomotion without a brain : physical reservoir computing in tensegrity structures. Artif. Life 19, 35–66 10.1162/ARTL_a_00080 23186351

[B6] Caluwaerts K. Schrauwen B. (2011). The body as a reservoir: locomotion and sensing with linear feedback, in Proceedings of the 2nd International Conference on Morphological Computation (ICMC 2011), (Venice), 45–47

[B7] Collins S. H. Wisse M. Ruina A. Tedrake R. (2005). Efficient bipedal robots based on passive-dynamic walkers. Science 307, 1082–1085 10.1126/science.1107799 15718465

[B8] Feinstein N. Nesher N. Hochner B. (2011). Functional morphology of the neuromuscular system of the octopus vulgaris arm. Vie et Milieu 61, 219–229

[B9] Franceschini N. Pichon J. M. Blanes C. Brady J. M. (1992). From insect vision to robot vision. Philos. Trans. Biol. Sci. 337, 283–294 10.1098/rstb.1992.0106

[B10] Füchslin R. M. Dzyakanchuk A. Flumini D. Hauser H. Hunt K. J. Luchsinger R. H. (2013). Morphological computation and morphological control: steps towards a formal theory and applications. Artif. life 19, 9–34 10.1162/ARTL_a_00079 23186344

[B11] Gutfreund Y. (1998). Patterns of arm muscle activation involved in octopus reaching movements. J. Neurosci. 18, 5976–5987 9671683 10.1523/JNEUROSCI.18-15-05976.1998 PMC6793066

[B12] Gutfreund Y. Flash T. Yarom Y. Fiorito G. Segev I. Hochner B. (1996). Organization of octopus arm movements: a model system for studying the control of flexible arms. J. Neurosci. 16, 7292–7307 8929436 10.1523/JNEUROSCI.16-22-07297.1996 PMC6578955

[B13] Hauser H. Ijspeert A. J. Füchslin R. M. Pfeifer R. Maass W. (2011). Towards a theoretical foundation for morphological computation with compliant bodies. Biol. Cybern. 105, 355–370 10.1007/s00422-012-0471-0 22290137

[B14] Hauser H. Ijspeert A. J. Füchslin R. M. Pfeifer R. Maass W. (2012). The role of feedback in morphological computation with compliant bodies. Biol. Cybern. 106, 1–12 10.1007/s00422-012-0516-4 22956025

[B15] Ijspeert A. J. (2001). A connectionist central pattern generator for the aquatic and terrestrial gaits of a simulated salamander. Biol. Cybern. 83, 331–348 10.1007/s004220000211 11357547

[B16] Jaeger H. (2002). Tutorial on training recurrent neural networks, covering bptt, rtrl, ekf and the “echo state network” approach. Technical Report 159, German National Research Center for Information Technology.

[B17] Jaeger H. (2003). Adaptive nonlinear system identification with echo state networks, in Advances in Neural Information Processing Systems, eds Becker S. Thrun S. Obermayer K. (Cambridge, MA: MIT Press), 593–600

[B18] Jaeger H. Haas H. (2004). Harnessing nonlinearity: predicting chaotic systems and saving energy in wireless communication. Science 314, 78–80 10.1126/science.1091277 15064413

[B19] Kandel E. R. Schwartz J. H. Jessell T. M. (2000). Principles of Neural Science. New York, NY: McGraw-Hill, Health Professions Division

[B20] Kang R. Branson D. T. Guglielmino E. Caldwell D. G. (2012). Dynamic modeling and control of an octopus inspired multiple continuum arm robot. Comput. Math. Appl. 64, 1004–1016 10.1016/j.camwa.2012.03.018

[B21] Kang R. Kazakidi A. Guglielmino E. Branson D. T. Tsakiris D. P. Ekaterinaris J. A. (2011). Dynamic model of a hyper-redundant, octopus-like manipulator for underwater applications, in Proceedings of 2011 IEEE/RSJ International Conference on Intelligent Robots and Systems (IROS), (San Francisco, CA), 4054–4059 10.1109/IROS.2011.6094468

[B22] Kazakidi A. Vavourakis V. Pateromichelakis N. Ekaterinaris J. A. Tsakiris D. P. (2012). Hydrodynamic analysis of octopus-like robotic arms, in Proceedings of 2012 IEEE International Conference on Robotics and Automation (ICRA), (Saint Paul, MN), 5295–5300 10.1109/ICRA.2012.6225037

[B23] Khalil H. K. (2002). Nonlinear Systems. Upper Saddle River, NJ: Prentice Hall

[B24] Kier W. M. Curtin N. A. (2002). Fast muscle in squid (loligo pealei): contractile properties of a specialized muscle fibre type. J. Exp. Biol. 205, 1907–1916 12077167 10.1242/jeb.205.13.1907

[B25] Kier W. M. Smith K. K. (1985). Tongues, tentacles and trunks: the biomechanics of movement in muscular-hydrostats. Zool. J. Linn. Soc. 83, 307–324 10.1111/j.1096-3642.1985.tb01178.x

[B26] Kuwabara J. Nakajima K. Kang R. Branson D. T. Guglielmino E. Caldwell D. G. (2012). Timing-based control via echo state network for soft robotic arm, in Proceedings of the 2012 International Joint Conference on Neural Networks (IJCNN), (Brisbane), 1–8 10.1109/IJCNN.2012.6252774

[B27] Laschi C. Mazzolai B. Cianchetti M. Margheri L. Follador M. Dario P. (2012). A soft robot arm inspired by the octopus. Adv. Robot. 26, 709–727 10.1163/156855312X626343

[B28] Laschi C. Mazzolai B. Mattoli V. Cianchetti M. Dario P. (2009). Design of a biomimetic robotic octopus arm. Bioinspir. Biomim. 4:015006 10.1088/1748-3182/4/1/015006 19258690

[B29] Li T. Nakajima K. Calisti M. Laschi C. Pfeifer R. (2012). Octopus-inspired sensorimotor control of a multi-arm soft robot, in Proceedings of 2012 International Conference on Mechatronics and Automation (ICMA), (Chengdu), 948–955 10.1109/ICMA.2012.6283271

[B30] Li T. Nakajima K. Cianchetti M. (2011a). Finding structure in deadtime, in Proceedings of the 2nd International Conference on Morphological Computation (ICMC 2011), (Venice), 47–49

[B31] Li T. Nakajima K. Kuba M. Gutnick T. Hochner B. Pfeifer R. (2011b). From the octopus to soft robots control: an octopus inspired behavior control architecture for soft robots. Vie et Milieu 61, 211–217 21670493

[B32] Li T. Nakajima K. Pfeifer R. (2013). Online learning technique for behavior switching in a soft robotic arm, in Proceedings of 2013 IEEE International Conference on Robotics and Automation (ICRA), (Karlsruhe), 1288–1294

[B33] Lieber R. (2002). Skeletal Muscle Structure, Function, and Plasticity: The Physiological Basis of Rehabilitation. Philadelphia, PA: Lippincott Williams and Wilkins

[B34] Lukoševičius M. Jaeger H. (2009). Reservoir computing approaches to recurrent neural network training. Comput. Sci. Rev. 3, 127–149

[B35] Maass W. Natschlaeger T. Markram H. (2002). Real-time computing without stable states: a new framework for neural computation based on perturbations. Neural Comput. 14, 2531–2560 10.1162/089976602760407955 12433288

[B36] Mazzolai B. Laschi C. Cianchetti M. Patane F. Bassi-Luciani L. Izzo I. (2007). Biorobotic investigation on the muscle structure of an octopus tentacle, in Proceedings of the 29th Annual International Conference of the IEEE EMBS, (Lyon), 1471–1474 10.1109/IEMBS.2007.4352578 18002244

[B37] Nakajima K. Hauser H. Kang R. Guglielmino E. Caldwell D. G. Pfeifer R. (2013). Computing with a muscular-hydrostat system, in Proceedings of 2013 IEEE International Conference on Robotics and Automation (ICRA), (Karlsruhe), 1496–1503

[B38] Nakajima K. Li T. Kang R. Guglielmino E. Caldwell D. G. Pfeifer R. (2012a). Local information transfer in soft robotic arm, in Proceedings of 2012 IEEE International Conference on Robotics and Biomimetics (ROBIO), (Guangzhou), 1273–1280 10.1109/ROBIO.2012.6491145

[B39] Nakajima K. Ngouabeu A. M. T. Miyashita S. Göldi M. Füchslin R. M. Pfeifer R. (2012b). Morphology-induced collective behaviors: dynamic pattern formation in water-floating elements. PLoS ONE 7:e37805 10.1371/journal.pone.0037805 22715370 PMC3371064

[B40] Nakajima K. Li T. Kuppuswamy N. Pfeifer R. (2011a). Harnessing the dynamics of a soft body with “timing”: octopus inspired control via recurrent neural networks, in Proceedings of 2011 15th International Conference on Advanced Robotics (ICAR), (Tallinn), 277–284

[B41] Nakajima K. Li T. Pfeifer R. (2011b). Timing and behavioral efficiency in controlling a soft body: a case study in octopus reaching behavior, in Proceedings of the 2nd International Conference on Morphological Computation (ICMC 2011), (Venice), 132–134

[B42] Nakajima K. Li T. Sumioka H. Cianchetti M. Pfeifer R. (2011c). Information theoretic analysis on a soft robotic arm inspired by the octopus, in Proceedings of 2011 IEEE International Conference on Robotics and Biomimetics (ROBIO), (Phuket), 110–117 10.1109/ROBIO.2011.6181271

[B43] Pfeifer R. Bongard J. (2006). How the Body Shapes the Way We Think: A New View of Intelligence. Cambridge, MA: The MIT Press

[B44] Pfeifer R. Lungarella M. Iida F. (2007). Self-organization, embodiment, and biologically inspired robotics. Science 318, 1088–1093 10.1126/science.1145803 18006736

[B45] Pfeifer R. Lungarella M. Iida F. (2012). The challenges ahead for bio-inspired ‘soft’ robotics. Commun. ACM 55, 76–87 10.1145/2366316.2366335

[B46] Proctor J. Holmes P. (2010). Reflexes and preflexes: on the role of sensory feedback on rhythmic patterns in insect locomotion. Biol. Cybern. 102, 513–531 10.1007/s00422-010-0383-9 20358220

[B47] Righetti L. Ijspeert A. J. (2008). Pattern generators with sensory feedback for the control of quadruped locomotion, in Proceedings of IEEE International Conference on Robotics and Automation (ICRA), (Pasadena, CA), 819–824

[B48] Schrauwen B. Verstraeten D. Campenhout J. V. (2007). An overview of reservoir computing: theory, applications and implementations, in Proceedings of the 15th European Symposium on Artificial Neural Networks, (Bruges), 471–482

[B49] Shepherd R. F. Ilievski F. Choi W. Morin S. A. Stokes A. A. Mazzeo A. D. (2011). Multigait soft robot. Proc. Natl Acad. Sci. U.S.A. 108, 20400–20403 10.1073/pnas.1116564108 22123978 PMC3251082

[B50] Shinohara M. Sabra K. Gennisson J. L. Fink M. Tanter M. (2010). Real-time visualization of muscle stiffness distribution with ultrasound shear wave imaging during muscle contraction. Muscle Nerve 42, 438–441 10.1002/mus.21723 20665510

[B51] Smith K. K. Kier W. M. (1989). Trunks, tongues, and tentacles: moving with skeletons of muscle. Am. Sci. 77, 28–35

[B52] Steltz E. Mozeika A. Rodenberg N. Brown E. Jaeger H. M. (2009). Jsel: jamming skin enabled locomotion, in Proceedings of IEEE/RSJ International Conference on Intelligent Robots and Systems (IROS), (St. Louis), 5672–5677

[B53] Sumbre G. Fiorito G. Flash T. Hochner B. (2005). Motor control of flexible octopus arms. Nature 433, 595–596 10.1038/433595a 15703737

[B54] Sumbre G. Gutfreund Y. Fiorito G. Flash T. Hochner B. (2001). Control of octopus arm extension by a peripheral motor program. Science 293, 1845–1848 10.1126/science.1060976 11546877

[B55] Sumioka H. Hauser H. Pfeifer R. (2011). Computation with mechanically coupled springs for compliant robots, in Proceedings of the IEEE/RSJ International Conference on Intelligent Robots and Systems (IROS), (San Francisco, CA), 4168–4173

[B56] Taylor J. Kier W. (2003). Switching skeletons: hydrostatic support in molting crabs. Science 301, 209–210 10.1126/science.1085987 12855806

[B57] Trivedi D. Rahn C. D. Kier W. M. Walker I. D. (2008). Soft robotics: biological inspiration, state of the art, and future research. Appl. Bionics Biomec. 5, 99–117 10.1080/11762320802557865

[B58] Vavourakis V. Bampasakis D. Kazakidi A. Pateromichelakis N. Ekaterinaris J. A. Tsakiris D. P. (2012a). Generation of primitive behaviors for non-linear hyperelastic octopus-inspired robotic arm, in Proceedings of IEEE RAS/EMBS International Conference on Biomedical Robotics and Biomechatronics (BioRob 2012), (Roma), 725–730

[B59] Vavourakis V. Kazakidi A. Tsakiris D. P. Ekaterinaris J. A. (2012b). A nonlinear dynamic finite element approach for simulating muscular hydrostats. Comput. Meth. Biomech. Biomed. Engin. [Epub ahead of print]. 10.1080/10255842.2012.723702 23025686

[B60] Verstraeten D. Schrauwen B. Haene M. D. Stroobandt D. (2007). An experimental unification of reservoir computing methods. Neural Netw. 20, 391–403 10.1016/j.neunet.2007.04.003 17517492

[B61] Yekutieli Y. Sagiv-Zohar R. Aharonov R. Engel Y. Hochner B. Flash T. (2005a). Dynamic model of the octopus arm. i. biomechanics of the octopus reaching movement. J. Neurophysiol. 94, 1443–1458 10.1152/jn.00684.2004 15829594

[B62] Yekutieli Y. Sagiv-Zohar R. Hochner B. Flash T. (2005b). Dynamic model of the octopus arm. ii. control of reaching movements. J. Neurophysiol. 94, 1459–1468 10.1152/jn.00685.2004 15829593

[B63] Zheng T. Branson D. T. Kang R. Cianchetti M. Guglielmino E. Follador M. (2012). Dynamic continuum arm model for use with underwater robotic manipulators inspired by octopus vulgaris, in Proceedings of 2012 IEEE International Conference on Robotics and Automation (ICRA), (Saint Paul, MN), 5289–5294 10.1109/ICRA.2012.6224685

